# Statistical and clustering analysis of attributes of Bitcoin backbone nodes

**DOI:** 10.1371/journal.pone.0292841

**Published:** 2023-11-08

**Authors:** Dawei Xu, Jiaqi Gao, Liehuang Zhu, Feng Gao, Jian Zhao

**Affiliations:** 1 School of Cyberspace Security, Beijing Institute of Technology, Beijing, China; 2 College of Cyber Security, Changchun University, Jilin, Changchun, China; Sunway University, MALAYSIA

## Abstract

Bitcoin is a decentralized digital cryptocurrency. Its network is a Peer-to-peer(P2P) network consisting of distributed nodes. Some of these nodes are always online and in this article are called Bitcoin backbone nodes. They have a significant impact on the stability and security of the Bitcoin network, so it is meaningful to analyze and discuss them. In this paper, we first continuously collect information about Bitcoin nodes from July 2021 through June 2022 (which is the longest duration of data collection to date). In total, we collect information on 127,613 Bitcoin nodes. At the same time, we conclude that the fluctuation of Bitcoin nodes is directly related to the fluctuation of onion network nodes. Further, we filtered 2694 Bitcoin backbone nodes based on our algorithm. By analyzing the backbone nodes’ attributes such as geographic distribution, client version, operator, node function, and abnormal port number, it is demonstrated that these nodes are centralized and play an important role in the Bitcoin network. Based on this, three unsupervised machine learning algorithms are selected to cluster multiple attributes of backbone nodes in a more scientific way. In this paper, the whole process from data collection to cluster analysis is completed and the best results are obtained by comparison. The experiments proved the existence of centralization of Bitcoin backbone nodes and obtained the number of nodes within each cluster. Finally, cluster nodes are de-anonymized based on the optimal results. Through our experiments, we obtain organizational information about the deployers of 103 nodes, linking the Bitcoin backbone nodes to the real world, thus accurately demonstrating the existence of Bitcoin centrality.

## Introduction

Bitcoin is the largest decentralized transaction system in terms of user volume and market value [1]. Since its proposal in 2008 [2], it has gained significant popularity. Due to its decentralized nature, it does not rely on any central authority for issuance and management, and transactions do not require intermediaries. Furthermore, anyone can run a client to join the system. However, the question remains: Is Bitcoin truly entirely decentralized?

Many studies have shown [3–5] that Bitcoin is not entirely decentralized, and the following centralization phenomena exist in practical applications. Firstly, there is a concentration of mining pools [6–8], as Bitcoin mining requires a significant amount of computing resources that are typically shared by miners in a pool. However, currently, there is a certain degree of centralization among Bitcoin mining pools, with a small number of pool operators controlling a large amount of computing power and having a certain level of control. Secondly, most Bitcoin transactions are completed through centralized exchanges [9–11]. These exchanges hold the assets and transaction records of users and possess certain supervisory and intervention capabilities. Thirdly, Bitcoin has a concentration of holders, with a relatively small number of individuals holding a significant amount of Bitcoin [12, 13]. The trading activities and holdings of these holders have a certain level of influence [14], leading to market fluctuations and price changes. Fourthly, despite the open-source development of Bitcoin [15, 16], only a few core developers actually have access to the code and update permissions. They have more control and influence compared to the normal user.

Although the above research can prove that Bitcoin nodes have a centralization phenomenon, it has the following shortcomings. 1. The correlation analysis has a lag. Since the increase in bitcoin price in 2020, the size of bitcoin nodes and the network topology relationship have changed significantly, but there is a lack of more recent correlation analysis of nodes. 2. Smaller amount of data. Existing analytical work on Bitcoin nodes mostly uses Bitcoin node data within a few weeks or months, so it is difficult to observe Bitcoin’s trend from the latitude of time. 3. Data statistics and analysis from a small view. Most of the existing analyses of node attributes are based on a single attribute and lack the ability to consider the relationship between nodes from multiple dimensions. We make up for the above shortcomings. Specifically, we verify the phenomenon of Bitcoin centrality using multi-feature, long-term time and large datasets. We demonstrate this in our data collection and analysis and Bitcoin backbone node clustering analysis.

We do more in-depth work on Bitcoin node detection and node clustering. In this paper, we obtain data through Bitnodes [17] (an open-source Bitcoin node detection tool, detailed in Data Collection). We use node detection tool Bitcoin Node Scanner [18] (BNS, detailed in Data Collection in this paper) to obtain data in large quantities, and use these data to make analysis and processing of Bitcoin nodes. The specific contributions are as follows:

1. We collect large amounts of Bitcoin network node data and analyze network node fluctuations. We make this data publicly available. This collection is the longest duration and largest amount of data in the last two years. Therefore the analysis is current.
2. We filter the Bitcoin network backbone nodes (nodes that have been online for a long time) from a large dataset. They play an important role in the Bitcoin network. We analyze the relevant properties of these nodes and summarize the common characteristics of such nodes.
3. We collect and analyze asset information of the backbone nodes of the Bitcoin network through a cyberspace search engine. For this asset information, we use unsupervised machine learning unsupervised algorithms to cluster them in multiple dimensions. We compare and select the optimal clustering results. Finally, we de-anonymize the backbone nodes based on the optimal results to get the real information of their deployers.

In this paper, Background and Related Work introduce the communication principle of Bitcoin nodes and the network topology. It also summarizes and compares the existing Bitcoin network analyses. Method summarizes this work’s primary contributions and design ideas. Data Collection and Analysis describe the data acquisition method and analyze the attributes of Bitcoin backbone nodes. Bitcoin Backbone Node Clustering Analysis and De-anonymization chapter clusters the various attributes of the Bitcoin backbone nodes and gives the true identity of the deployer.

## Background and related work

### Bitcoin network communications

The Bitcoin network runs three protocols, the Bitcoin protocol, the Pool mining protocol, and the Stratum protocol, of which the Stratum protocol is used for mining, lightweight Bitcoin wallets. In this paper, we focus on the Bitcoin protocol.

Bitcoin is a decentralized network topology. When a new network node starts and wants to join the network, the new node must discover the other nodes in the network and establish a connection with it. A new node joins in 2 steps. 1. The new node starts by sending a request to the seed node. This request fetches other nodes in the Bitcoin network (the seed node is hard-coded into the Bitcoin client program). 2. The new node sends an unsolicited connection request to other nodes. The other peer nodes communicate with the new node after receiving the connection request from the new node. The node information of the two successfully connected nodes is placed in their respective addr database(This database holds the addresses of bitcoin nodes that have communicated with it.) for the next connection or propagation to other nodes.

**Fig 1:**
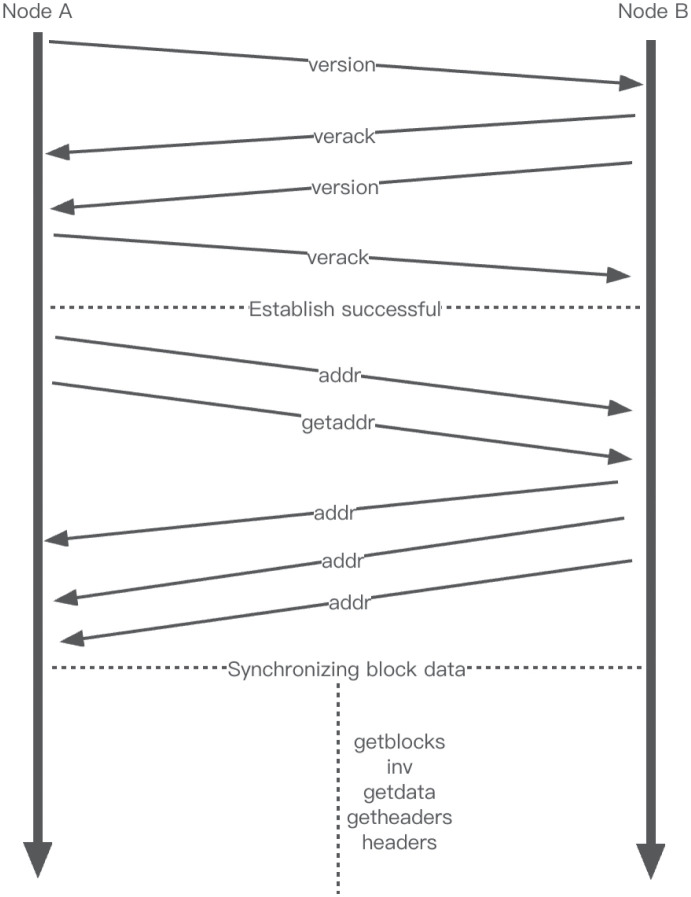
Bitcoin peer node communication flow.

As depicted specifically in Fig 1. Step 1: Node A will send a version message to its peer node B. This content contains the client’s version number, timestamp, and other information (version-specific structure [19]). Step 2: Node B will reply with a versionack to acknowledge receipt. Step 3: Node B will send a version message to node A (which contains the client information of node B). Step 4: Node A replies with a versionack indicating receipt. After the above process, the two nodes formally establish the connection.

After a successful connection, both nodes start exchanging the Bitcoin addresses in the addr database to update the new network topology. Step 1: Node A sends an addr message and a getaddr request to Node B. The getaddr request is used to obtain the IP address in Node B’s addr database. Step 2: Node B selects the IP address from the addr database and sends it to Node A. The selection is done by randomly picking the IP with the newer timestamp (a Bitcoin node has at most 8 output connections and 117 input connections [20]). Step 3: Node A and node B send getblock,inv,getdata,getheaders,headers [21] to each other to synchronize their data. Since the third step is not the focus of this paper, it is not introduced too much.

### Bitcoin network topology

P2P network communication architecture is used by the Bitcoin network. In a P2P network, each node uses the Bitcoin network services while providing services to other nodes [22].

**Fig 2:**
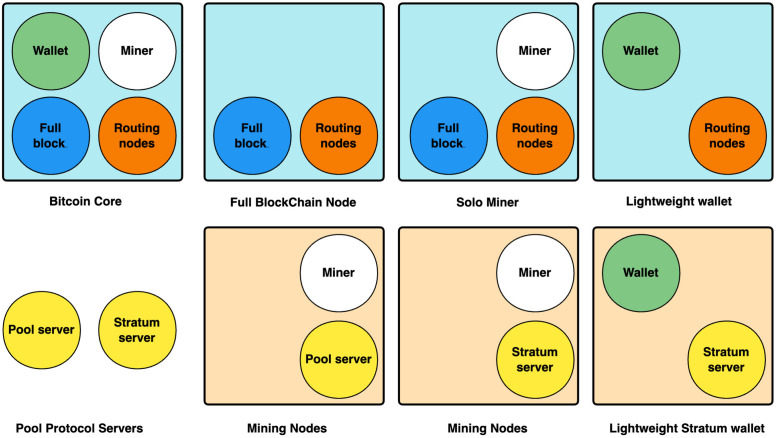
Bitcoin node types. The four pictures in the first row of the figure indicate the types of nodes running the Bitcoin protocol. The second row of four pictures indicates the type of node running the mining protocol.

The functionality of each node varies slightly. According to Fig 2, these include 1) Bitcoin core nodes, which have the full functionality of Bitcoin. 2) Bitcoin full nodes, which do not have mining or wallet functionality, but will sync all block data. 3) Independent miner nodes, which do not have wallet functionality and can mine alone. 4) Lightweight wallet nodes, which tend to be more numerous, do not sync all block data to save storage space, need to depend on other full nodes to verify transactions, and are suitable for ordinary users.

To secure the reliability of their own revenue, mining-focused nodes will join mining pools [23]. The nodes in the mining pool will each run a different mining protocol, such as the stratum protocol and the pool mining protocol.

**Fig 3:**
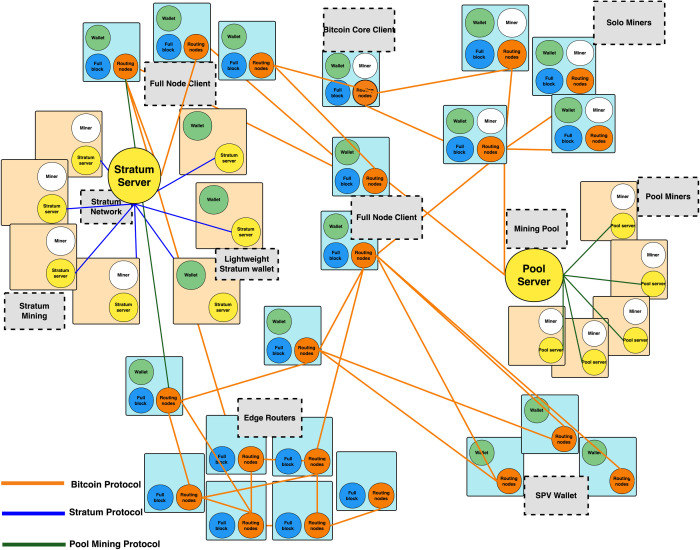
Bitcoin network topology.

Fig 3 displays the network topology of the Bitcoin network. Three protocols are used for communication on the Bitcoin network. We don’t study nodes running the Pool mining protocol or the Stratum protocol in this work. Instead, we concentrate on nodes running the Bitcoin protocol (nodes connected by yellow lines) [24]. Among them, the full-node client and the Bitcoin core client play an important role in the stability of the network.

### Related work

In the area of Bitcoin networks and de-anonymization, there have been many papers published by scholars in recent years. There are two directions based on the Bitcoin hierarchy. (1) Topology analysis and topology restoration based on the Bitcoin network layer. (2) Address de-anonymization clustering analysis based on the Bitcoin transaction layer. The former obtains the IP addresses of Bitcoin nodes and related client information by constructing Bitcoin network requests. The above information can analyze the network state. The latter collects Bitcoin network transaction addresses. Using clustering and other methods, it is possible to infer which anonymous user initiated the transaction. This paper combines the work of (1) and (2). We collect information about the Bitcoin network layer and analyze it for clustering and de-anonymization.

Based on the data analysis of the Bitcoin network layer, a relatively complete analysis was given by SEHYUN PARK. In this article, SEHYUN PARK collected Bitcoin data for 37 days in 2018 and counted the number of users, transactions, and prices of the Bitcoin system. The bitcoin node propagation mechanism is also analyzed [22]. The research in this paper is similar to SEHYUN PARK, but it is older and has few dimensions to analyze. The paper has the advantage of being current by analyzing the current, newer Bitcoin network in multiple dimensions.

During the analysis of the Bitcoin network, Bitcoin network fluctuations and node churn directly affect the system stability. In 2019 Muhammad Anas Imtiaz et al. of Boston University analyzed the impact of user churn on block propagation latency. Muhammad Anas Imtiaz et al. showed that user churn leads to an average 135% increase in block propagation time (i.e., 336.57 ms vs. 142.62 ms), which in the worst case may lead to an increase of up to 800 times [25].

In real networks, many Bitcoin network nodes are hidden behind Network Address Translation(NAT) or firewalls. Such nodes can usually actively connect to Bitcoin nodes, but they do not provide services to other Bitcoin nodes. These nodes are called unreachable nodes. The scheme proposed by Gengxian Li et al uses a structured P2P overlay network to store the index of unreachable nodes across the network and finally verifies the effectiveness of each module at different network sizes by deploying a realistic cluster environment [26]. In 2019, Ieryam Essaid published a system for dynamically detecting the topology of the Bitcoin network. The best feature of this system is the ability to analyze the Bitcoin network in real-time [27]. Meanwhile, Escobero Hernández and Guillermo analyzed the Bitcoin network characteristics. They found that the number of active nodes is 7530 and only 84.25% of the nodes can be connected, indicating the existence of some nodes that do not want to be connected from outside [28].

Wahrstätter et al use clustering algorithms to analyze the Bitcoin network in order to cope with the illegal transactions present in Bitcoin, which can detect the illegal behavior of users and effectively combat the crime [29]. However, we improve on Wahrstätter’s work by introducing a clustering analysis of Bitcoin network asset attributes. We found a correlation between attributes in the cluster analysis of Bitcoin backbone nodes. In 2019, Neudecker analyzed the characteristics of bitcoin nodes from 2015–2018 in terms of number of connectable nodes, network churn, an IP address belonging to the region, node latency, and other related attributes [30]. Neudecker in 2018 discussed that an unconstrained blockchain network should have strong performance, low participation cost, high anonymity, DoS resistance, and good network topology hiding ability. This criterion to quantify different types of blockchain networks illustrates that an unconstrained blockchain should satisfy the above characteristics [31].

## Method

To study whether there is a centralization problem in the Bitcoin system, in this paper, we analyze it through 4 steps. 1. Data collection. 2. Data filtering 3. Attribute analysis 4. Clustering and de-anonymization analysis. The first thing to do is data collection. Due to the decentralized nature of Bitcoin, the number of nodes is constantly changing, so it is important that the data is real-time. However, early work on Bitcoin node analysis suffers from long intervals, a small number of nodes, and short collection time. We collect continuous Bitcoin node information from July 2021 to June 2022 to compensate for these shortcomings.

The data needs to be further filtered. As shown by the Bitcoin Network Topology, most are edge nodes and a few are backbone nodes. Backbone nodes are stable online for a long time and have important functions such as verifying transactions, and storing ledgers for mining and are widely spread in the Bitcoin network. We prove that these nodes are controlled by a few organizations or individuals behind them. So screening and analyzing these nodes is important for us to analyze the Bitcoin centralization problem.

For the acquired bitcoin network backbone nodes, the first thing is the node attribute analysis. Picturing the geographic location attributes enables us to determine more intuitively whether their geographic location is centralized. The analysis of the client version can help us determine how updated and active the node is. The analysis of the operator can help us determine the type of terminal device of the node and whether there is a 51% attack problem. Node functionality analysis allows us to understand what functions they have turned on and verify that these Bitcoin nodes are backbone nodes.

To further validate the centralization problem, we introduce a cyberspace search engine for asset acquisition of bitcoin IP addresses and further cluster the backbone nodes by asset attributes. Whether certain nodes are related or not is considered from a social engineering perspective. The nodes are identified as belonging to a class by abnormal port numbers and services opened by the nodes. Machine learning clustering algorithms are introduced to cluster and analyze Bitcoin IP addresses by constructing asset attribute feature datasets. This clustering method takes into account multiple attributes among nodes and is more objective and universal. For the clustered data, we select the ones with the better results for de-anonymization analysis. Specifically, we analyze the relevance of web pages and associate them with real-world organizations or institutions. The final goal is de-anonymization.

## Data collection and analysis

### Data collection

We collected data primarily through two methods:

**Method 1**. Bitnodes is an open-source platform that focuses on detecting the Bitcoin network. It estimates the relative size of the Bitcoin P2P network by collecting information on reachable Bitcoin network nodes via a vast number of globally deployed distributed probe nodes. To obtain a batch of Bitcoin nodes, these probe nodes first send getaddr requests to a set of seed nodes. The platform then sends getaddr requests to the newly obtained Bitcoin nodes recursively until all reachable Bitcoin nodes are accessible. Moreover, these probe nodes send inv, getdata requests, and others to obtain detailed information about Bitcoin nodes. Finally, this information is stored in a database in the form of a network snapshot and updated every 5 minutes. We utilized Python 3.8 to develop web crawlers, using libraries such as requests and thread to retrieve official open-source data in bulk.

**Method 2**. Develop and implement BNS, a bitcoin network probing program written in Java, to recursively discover network node information through the netty framework and a multi-threaded mechanism. The program first dnsseed sends and obtains a Bitcoin node IP address request. The obtained address is saved in the queue, and then the IP address in the queue is used as the Bitcoin node IP address for secondary requests. Obtain the bitcoin nodes’ neighbor addresses, then add the result to the end of the queue. Repeat the above steps for bulk and efficient acquisition, the program will be open source.

Table 1 compares BNS with other Bitcoin node collection tools, which demonstrates the efficiency of our BNS tool in detecting Bitcoin nodes and its ability to collect information on unreachable Bitcoin nodes.

**Table 1:** Tool comparison result.

Node probe tool	Time Cost	Total	Reachable node
Bitnodes	5 min	11188	11188
BNS	3hours	4053015	10201
bitcoin-sniffer [32]	35days	782423	6913
blockchair [33]	-	7325	7325
bitrawr [34]	5days	17,460	17,460

We collect continuous Bitcoin network snapshot information from July 2021 to June 2022 through Method 1. The bitcoin timestamp information is 1626354450 to 1652763023, totaling 71101 snapshot data, and a total of 127613 IP addresses appear in the snapshots. The number of nodes in each snapshot is also counted, which contains a large number of onion network nodes. Meanwhile, the work in this paper focuses on the integration and analysis of the data.

**Fig 4:**
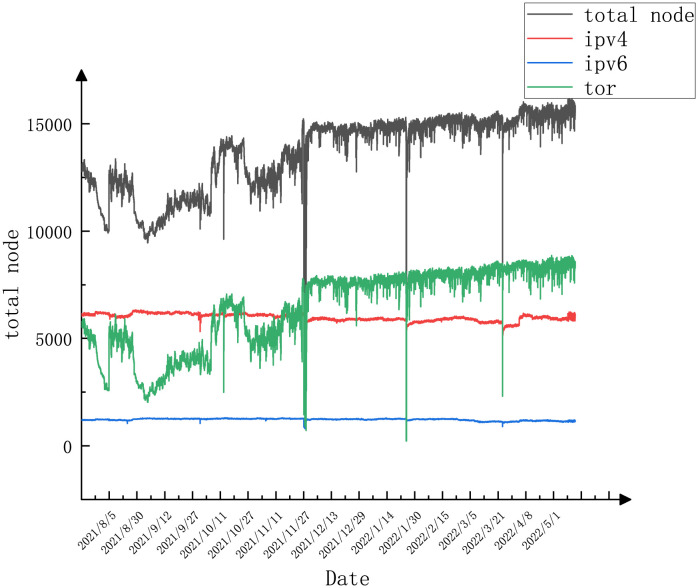
Change in the number of bitcoin nodes (202107–202206).

In this paper, we count the changes in the number of nodes over this period of time, and this behavior can reflect the trend of the Bitcoin system and its stability to some extent. The change of nodes over time is shown in Fig 4. There are three main types of Bitcoin network addresses, ipv4, ipv6, and tor (ending with.onion). The number of ipv4 and ipv6 nodes in the Bitcoin network is relatively stable and does not change much.

It is concluded that the fluctuation of bitcoin network nodes is mainly related to the number of tor nodes, and the number of tor nodes has been on a steady upward trend during the period of 2021–2022.

Method 2 complements the Method 1 dataset. It is used to detect the latest neighbor relationships between network nodes, and 490 stable nodes of bitcoin nodes are obtained by sending the getaddr request described in the Bitcoin Network Communications subsection by Method 2. A total of about 20w Bitcoin node addresses were obtained after 3 days of continuous listening. Why does method 2 find a large number of Bitcoin addresses compared to method 1?

The reasons for this are as follows. Bitcoin network node communication is bidirectional (node A actively sends messages to node B, while node B also responds to node A’s requests). Method 1 sends the constructed Bitcoin protocol to the node, gets the response from the node, and records the data. However, a large number of nodes in the network exist behind NAT networks or firewalls, and while they can actively connect to other nodes, other nodes cannot connect to them. This type of node cannot be captured by method 1. Compared to method 1, method 2 can obtain the bitcoin addresses in the **addr** database in the node, which contains addresses that cannot be actively connected. So the number of addresses captured by method 2 is much larger than that of method 1. However, these large number of addresses, which only indicate that they were once connected to the Bitcoin network, need to be further filtered.

### Data analysis

**Fig 5:**
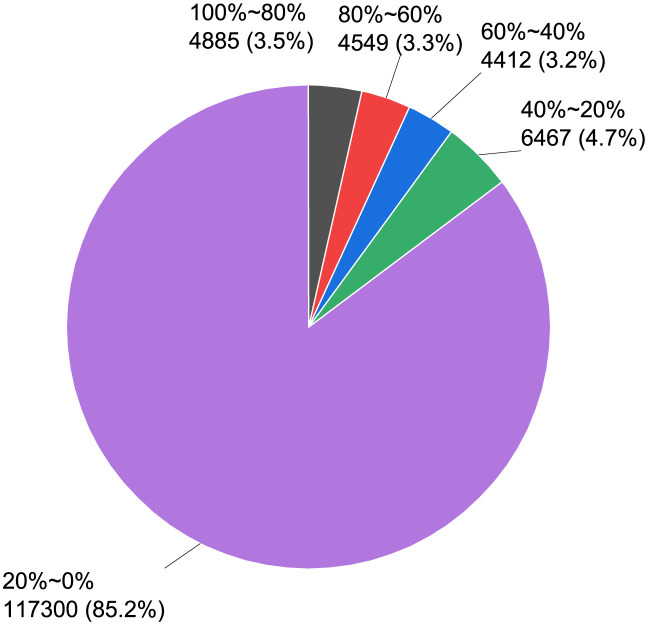
Node online rate number percentage chart.

We presume that if a Bitcoin IP address appears in two consecutive snapshots, it is assumed to have been active for the full five minutes. We calculate the online rate of the nodes in the snapshot from July 2021 to June 2022. Fig 5 shows the statistical graph with a 20% interval. In this paper, we find that 85.2% of the nodes are online rate at less than 20%.

According to the Bitcoin network topology chapter, we collect nodes belonging to the yellow connected nodes in Fig 3. These data contain Bitcoin core nodes, Bitcoin full nodes, independent miner nodes, and lightweight wallet nodes. Most of these nodes belong to individual user identities and are edge nodes in the Bitcoin network. Their joining and leaving will not have an impact on the Bitcoin network, so we focuse on analyzing the Bitcoin network backbone nodes. Therefore, we obtain the Bitcoin network backbone nodes by using Algorithm 1.

**Algorithm 1** Get Backbone Nodes

**Input**: *Normal*_*Nodes*

**Output**: *BackboneNode*

1: **for** Normal_Node ∈ Normal_Nodes[1,…,j] **do**

2:  Count = 0

3:  **for** Snapshot ∈ Snapshots[1,…,n] **do**

4:   **if** Normal_Node exist in Snapshot **then**

5:    Count ⇐ Count +1

6:   **end if**

7:  **end for**

8:  **if** Count/n ≥ 90% **then**

9:   Normal_Node is Backbone Nods

10:  **else**

11:   Normal_Node is not Backbone Nods

12:  **end if**

13: **end for**

In this paper, nodes with an occurrence rate greater than 90% in the snapshot are considered backbone nodes. Considering the network churn, network latency and other instability factors, Bitcoin nodes are hardly 100% online. According to the statistics, among 127613 bitcoin nodes, the number of backbone nodes that satisfy the above filtering criteria is 2694, among which there are 114 onion network nodes.

These nodes satisfy the following principles.

**Principle I**. Not an Normal User. The normal Bitcoin user is in demand for trading purposes and disconnects immediately when the transaction is completed. Meanwhile, most of the normal users use lightweight wallets that do not synchronize local data in order to save space, and most of them use cell phones, personal computers, and other terminal devices. These devices typically rely on the cooperation of Bitcoin exchanges or full nodes, which do not have long-term online conditions. The 2,694 bitcoin backbone nodes mentioned above have been operating for close to a year and are responsive to external nodes. Most of them are long-term online devices such as cloud servers and mining nodes.

**Principle II**. Undertake important functions. Although Bitcoin nodes are peer nodes, as known from the introduction in the Bitcoin Network Topology subsection, a large number of nodes are edge nodes. Based on the communication mechanism of the Bitcoin network, peer nodes will periodically send keep-alive requests to neighbor nodes, if the neighbor nodes do not respond, they will be placed at the bottom of the address pool if the neighbor nodes give a response, the next communication will be priority connection, priority forwarding, and follow the “fittest-gets-richer” phenomenon (the more important nodes can get more benefits) [35]. The 2694 long-term online nodes mentioned above are active nodes that respond to requests in a timely manner, and thus these nodes play an important role in Bitcoin network communication.

**Geographical distribution**.

**Fig 6:**
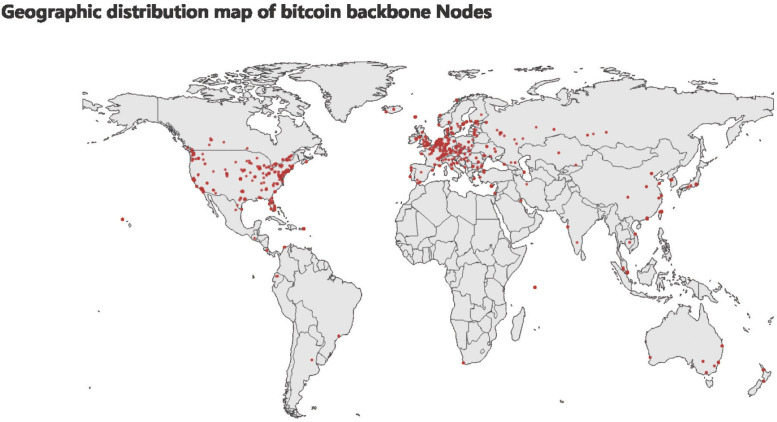
Geographical distribution of backbone nodes.

For the above-filtered backbone nodes, we draw a map based on their latitude and longitude information (Fig 6).

**Conclusion**. Most of the Bitcoin backbone nodes are located in Europe and the United States. There is some degree of geographic centralization. South America and Africa generally have fewer backbone nodes.

We counted the more important attributes of these backbone nodes, such as client versions, operators, and node functionality. The analysis of these attributes helps to understand the composition of the Bitcoin network and also helps to regulate the Bitcoin network.

**Client versions**.

**Fig 7:**
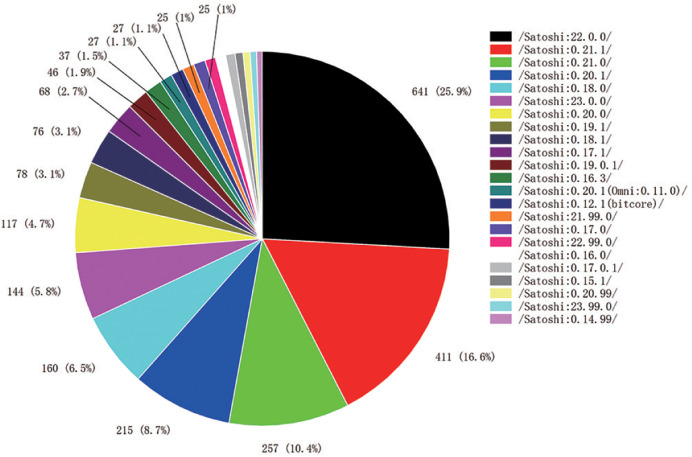
Percentage of Bitcoin by protocol version.

Satosh: 22.0.0 accounts for nearly 1/4 of the Bitcoin client versions (Fig 7). This version is the first major release to support the Bitcoin Taproot protocol, and subsequent versions no longer begin with 0.x. This version, released on September 13, 2021, removes the anonymous browser TorV2 version and now supports the TorV3 version and Invisible Internet Project (I2P) [36], among others.

**Conclusion**. Nearly 32% of the backbone nodes are maintained for a long time and are updated in a timely manner, which plays an important role in the versioning and system development of the Bitcoin network. From the percentage of client protocol versions collected, a partial centralization of client versions is observed.

**Operators**.

The analysis of the operators allows further clustering of the Bitcoin network and determines if there is currently a 51% attack problem on Bitcoin nodes. According to the statistics, there are 459 different operators, with the larger ones being the data center operator in Gunzenhausen, Germany, OVH Groupe SAS cloud computing company in France, digital ocean cloud hosting merchant in the US, Amazon in the US, Alibaba in China, and Onion Network. None of these cloud service vendor nodes account for more than 50% of the total.

**Fig 8:**
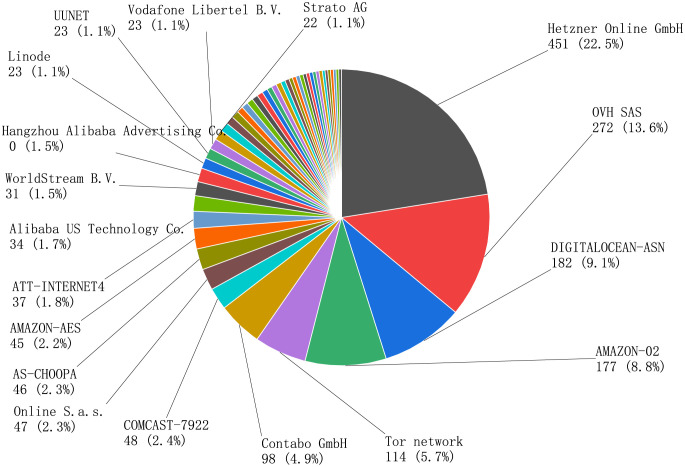
Bitcoin network affiliated operators.

**Conclusion**. The backbone nodes are predominantly cloud servers, proving principle I. (as shown in Fig 8).

**Node Function**.

As can be seen from the Bitcoin Network Topology subsection, Bitcoin nodes can be classified into different types of nodes depending on their functions. The following shows the functions that the nodes have and the numbers that can represent them.

**NODE_NETWORK (1)**: The node can provide complete blocks instead of just block headers, and the node is the core client of Bitcoin.

**NODE_GETUTXO (2)**: The node supports getutxo protocol requests, which are not supported by the Bitcoin Core client.

**NODE_BLOOM (4)** The nodes are capable and willing to support Bloom filters. The Bitcoin Core client supports this feature by default without announcing the number.

**NODE_WITNESS (8)**: The node can respond to block and transaction requests that contain isolated witnesses.

**NODE_XTHIN (16)**: discontinued. The node supports Xtreme Thinblocks.

**NODE_COMPACT_FILTERS (64)**: Indicates that the block filter index of the node catches up with the active chain.

**NODE_NETWORK_LIMITED (1024)**: Basically equal to NODE_NETWORK, but only provides information about 288 blocks, i.e. blocks within 2 days.

**Fig 9:**
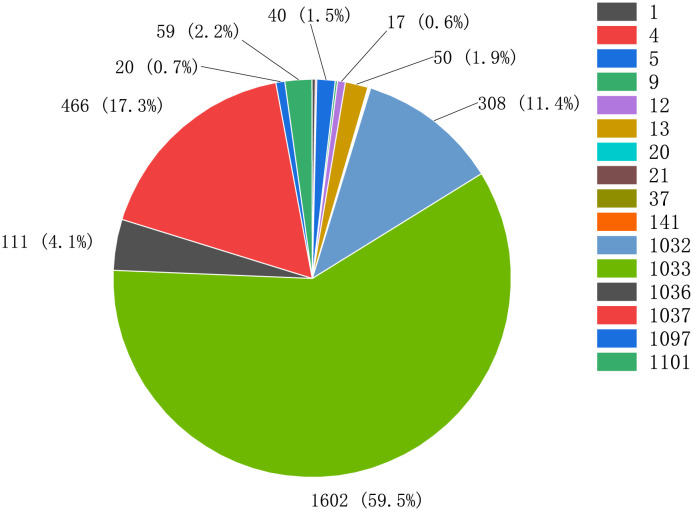
Bitcoin backbone node function statistics. The numbers on the right side of the figure represent the different functions of the nodes. As an example: 1101 (Simultaneous functions) = 1024 (NODE NETWORK LIMITED) + 64 (NODE COMPACT FILTERS) +8 (NODE WITNESS) + 4 (NODE BLOOM) + 1 (NODE NETWORK).

**Conclusion**. Backbone nodes assume several important functions (Fig 9), with the largest percentage of nodes having the most open functions, validating principle II and indicating the important role of backbone nodes in the Bitcoin network. These nodes are open to NODE_NETWORK_LIMITED, NODE_COMPACT_FILTERS, NODE_WITNESS, and NODE_NETWORK. Additionally, these nodes typically store a large amount or all of the ledger data and provide data synchronization to light nodes. Wallet nodes provide the function of verifying transactions.

## Bitcoin backbone node clustering analysis

We collected and analyzed the data of Bitcoin nodes in Data Collection and Analysis, and concluded that Bitcoin backbone nodes play an important role in the Bitcoin network. Most of these backbone nodes belong to organizations or groups, but they have no corresponding real-world organizations. In this chapter, we further research the similarity between nodes and which organizations in the real world correspond to nodes.

### Attribute acquisition of node assets

In order to obtain better and more comprehensive Bitcoin node attribute information, we introduce a new data collection method, which is based on the node asset attribute collection method of the cyberspace search engine.

Cyberspace search engine [37] is a search engine for geek groups. Different from traditional search engines such as Google and Yahoo, cyberspace search engines can detect a wider range of devices and have more comprehensive content. The main detected devices include PCs, servers, routers, IoT home appliances, and even monitoring probes and sensitive devices in the industrial control field. These devices are collectively considered nodes. The cyberspace search engine analyzes the characteristics of the above-mentioned nodes through the back-end distributed crawler and obtains information such as IP addresses, port numbers, and geographical locations of these networked devices.

We use zoomeye [38] to obtain asset information. Algorithm 2 describes the specific process. Because of the limitation of the network space search engine, the onion node does not have a good effect. So we only analyze ipv4 and ipv6 type addresses. The cyberspace search engine will periodically crawl to the nodes in the network and save them as snapshots. In order to ensure the effectiveness and availability of the data, we only select the snapshot information after January 1, 2022, as the Bitcoin node attributes.

**Algorithm 2** Obtain node attribute information through cyberspace search engine

**Input**: bitcoin_nodes[1,…,18887]

**Output**: bitcoin_nodes_attribute[]

 **for** node ∈ bitcoin_nodes[1,…,18887] **do**

2:   **if** node ∈ tor_network **then**

    continue

4:   **else**

    node_property,timestamp ⇐ search_by_cyberspace(node)

6:    **if** timestamp ≥ 2022/01/01 **then**

     bitcoin_nodes_attribute[] ⇐ save_bitcoin_nodes_attribute(node_attribute)

8:    **end if**

  **end if**

10: **end for**

 return bitcoin_nodes_attribute[]

### Cluster analysis based on abnormal port numbers

According to the official data of Bitnodes on July 2, the proportion of nodes with open port 8333 is 94.36% [39]. There are two main reasons: 1. The default port number of the Bitcoin client is 8333. 2. The default port is easier to find. When users run the Bitcoin client, they not only need to discover other Bitcoin nodes but also need to spread their own information to the network. Opening port 8333 means that its information can be obtained by various port scanning software, and these nodes are also willing to provide services for other nodes.

From the perspective of social engineering, we regard nodes with ports other than 8333 as nodes with abnormal port numbers. These nodes are usually unwilling to be found. The deployed subjectively customizes a port number by modifying the Bitcoin client configuration file. Therefore, we have reason to believe that when two or more nodes with the same abnormal port number appear at the same time, the probability that these two nodes belong to the same organization or group behind them is relatively high.

We collected and sorted out the abnormal port numbers of the backbone nodes, classified them, and judged their internal relationships. According to statistics, there are a total of 64 different types of nodes with non-8333 port numbers, and we classify nodes belonging to the same port number into one category.

In order to verify the accuracy of the clustering, we conduct further analysis on the nodes of the same class. Therefore, the latitude and longitude of the node and the attribute of the operator are introduced to verify our idea. According to the analysis of the clustering results in Table 2 (See S1 Table for the complete table), we found that the latitude and longitude information of the nodes with the same abnormal port number is very close, and even most of the nodes have the same geographic location. The similarity of operator attributes of nodes of the same type reaches more than 85%.

**Table 2:** Cluster analysis results of abnormal port numbers (partial).

ip	port	lon	lat	organization
221.219.97.105	2001	116.397459	39.938884	China Unicom Beijing Province Network
221.219.102.228	2001	116.397459	39.938884	China Unicom Beijing Province Network
63.32.65.205	5001	-6.26031	53.349805	Amazon.com, Inc.
54.76.224.236	5001	-6.26031	53.349805	Amazon.com, Inc.
34.248.223.238	5001	-6.26031	53.349805	Amazon.com, Inc.
34.252.91.3	5001	-6.26031	53.349805	Amazon.com, Inc.
54.195.171.69	5001	-6.26031	53.349805	Amazon.com, Inc.
213.239.232.113	8332	11.07349	49.454342	Hetzner Online GmbH
163.172.142.149	8332	2.352222	48.856614	ONLINE S.A.S.
162.55.101.8	8332	11.07349	49.454342	Hetzner Online GmbH
162.55.7.43	8332	12.364975	50.475005	Hetzner Online GmbH

**Fig 10:**
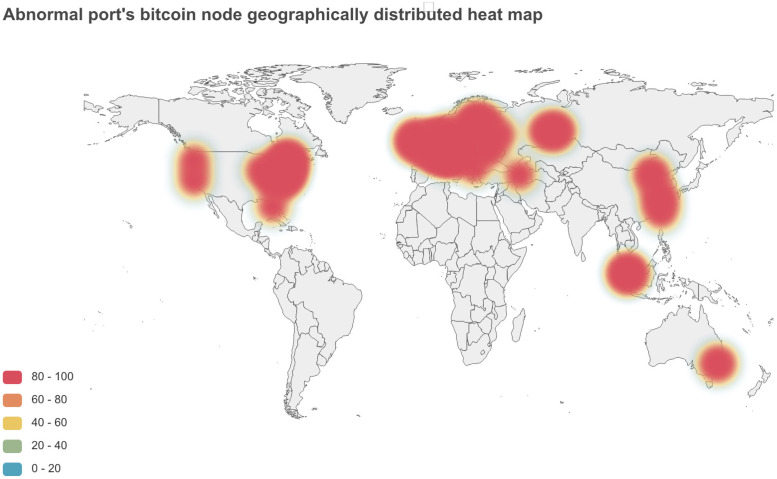
Heat map of abnormal port number node distribution. The larger the red area is, the greater the number of abnormal nodes.

We visualize the clustering results. According to its latitude and longitude information, the clustering results are mapped to the world map. Fig 10 below shows the heat map of the geographical distribution of abnormal port numbers. The red area represents the number of abnormal port numbers.

We conclude that nodes with the same abnormal port number, the same or similar latitude and longitude, and the same operator are strongly correlated nodes. We think they belong to the same organization or group behind them.

### Analysis based on service type

From the backbone nodes obtained in Chapter Data Collection and Analysis, it can be known that this long-term online node not only has multiple important functions in the Bitcoin node function (mentioned in Bitcoin Network Topology), but also provides other services to the real world. For example: Bitcoin exchanges OKEX, Huobi, Binance, etc [10] will deploy a large number of full nodes (backbone nodes in this paper). These nodes provide wallet services to registered users. The user’s wallet synchronizes block data and verifies transactions by connecting to these nodes. Some nodes are network probe nodes. When providing network communication, they will record the nodes that have been connected. Nodes will save these data for Bitcoin network analysis. Some nodes act as online Bitcoin ATMs, and users access these nodes to conduct Bitcoin transactions.

We use the cyberspace search engine to obtain the asset information of the backbone nodes, further determine which services are opened by these nodes, and classify the services. Specifically, we count whether the web service of the node is open, what functions the web service provides, and services such as FTP, SSH, and domain. We believe that nodes with the same web framework, the same website interface, and the same return status code belong to one type of node. We analyze which institutions and organizations in the real world the website corresponds to, so as to de-anonymize the Bitcoin nodes.

According to statistics, among the 2117 ipv4 addresses, 2045 open services, account for 96.6%, most of which are HTTP/HTTPS. Table 3 shows the statistical results of some open service types. Since most servers have opened both HTTP service and HTTPS service, the number of acquired addresses will be more than the number of ipv4 addresses.

**Table 3:** Bitcoin backbone node open services (partial).

Service	HTTP	HTTPS	bitcoin	SSH	SMTP	IMAP	FTP	POP3	domain	MySQL
**Count**	3303	3030	1919	1230	370	284	198	171	135	128

We conclude that the backbone nodes of the Bitcoin network not only play an important role in the communication of the Bitcoin network but also provide different kinds of Bitcoin services to the outside world. They belong to the “fittest-gets-richer” type of node described in Section Data Analysis. HTTP and HTTPS are relatively characteristic services and the services with the highest proportion. Next, we will obtain and analyze the specific information of the web service. These web services are mainly divided into eight categories: 1. Wallet websites. 2. Bitcoin node monitoring website. 3. Bitcoin transaction monitoring website. 4. Bitcoin data file download service. 5. Mining pool performance detection and mining pool information. 6. Ordinary empty pages. 7. User background login interface. 8. Mining information interface. Part of the interface is given in S1 Fig.

### Clustering analysis of bitcoin addresses based on machine learning

By analyzing the above attributes, cluster analysis is performed manually according to some attributes. However, comprehensive analysis based on all attributes also needs to be considered. We want to explore the relationship between data and data through machine learning methods, which makes the results more theoretical and scientific. Through unsupervised algorithms, clustering algorithms can help us analyze and de-anonymize bitcoin nodes. It should be noted that the evaluation of unsupervised clustering algorithms is a challenging task. There is no clear evaluation standard for explaining the results, and the general trend of the data can only be analyzed as a whole.

**The first step**: data collection.

In order to make the machine learning clustering effect more accurate, we expand the clustering dataset. First, through Algorithm 1, the online rate of nodes is set to be greater than 20%, and a total of 20,313 Bitcoin nodes are selected as original nodes. Then obtain its node attribute information through Algorithm 2. After deletion and selection by Algorithm 2, there are 9651 remaining nodes, all of which are of type ipv4 and ipv6, and do not contain onion nodes. Table 4 below shows the initial feature information of the acquired data.

**Table 4:** Bitcoin backbone node open services (partial).

feature	description(data type)
IP	Bitcoin node IP address (string)
port	open port (int)
protocol version	Bitcoin P2P network protocol version (int)
user-agent	Bitcoin client version (string)
service	bitcoin node type (int)
connected since	last connection time (int)
height	Current block height (int)
host name	Bitcoin node hostname (string)
City	City (string)
country code	country code (string)
lat	The latitude of the node (float)
lon	The longitude of the node (float)
Timezone	The time zone where the node is located (string)
ASN	Autonomous system number (string)
organization	Belonging running information (string)
snapshots count	the number of times to appear in the snapshot (int)

**The second step**: data preprocessing.

Thinking from the perspective of data correlation, first, remove the strong correlation features. Since the **organization** and **ASN** features are not independent of each other, only the ASN is selected as a feature. (**lon, lat**) and **city** belong to the inclusion relationship, **city** and **country code** belong to the inclusion relationship, and **Timezone** and **city** also belong to the inclusion relationship, so only **city** is selected as the feature. Also remove some features that are not relevant to clustering, for example: **height**, **IP**, and **connected since**. Since the **snapshot count** can intuitively reflect the online rate of the node, it is reserved. The last reserved features are **port**, **protocol version**, **user agent**, **service**, **city**, **ASN**, and **snapshot count**.

After selecting the feature, the missing values in the feature need to be filled in. Among the above-reserved features, the **city** and **ASN** fields are partially missing. In order to fill in the missing fields, we uses third-party tools to query and fill in. The specific APIs are https://www.ip2location.io/ and https://www.ipvoid.com/ip-to-asn/.

Since snapshot counts have many values that are not suitable for clustering, the data values need to be generalized. We classify the numerical features of the snapshots count according to the line rate, and generalize the features into snapshots online rate features such as 0%–20%, …, 80%–100%.

**The third step**: feature encoding.

String-type features usually need to be converted into numerical features in machine learning algorithms to have a better clustering effect. Therefore, it is necessary to encode the features in the data set. Commonly used feature encoding methods include Binary Encoding, Hashing Encoding, Count Encoding, and Embedding Encoding. Since there are many feature values of the **user agent**, **city**, and **ASN** in this data set, Binary Encoding will lead to an explosion of feature latitude. Count Encoding has a certain probability of encoding collisions, so it is not applicable to this article. An encoding collision is when two or more different categories may have the same count, resulting in them having the same value after encoding. However, Embedding Encoding will compress features and result in a decrease in clustering accuracy. In the end, we choose the Hash Encoding, it follows that

$$\begin{array}{c} hash\left(x\right)=hash\_function\left(x\right)mod2^{d}. \end{array}$$ (1)

We convert the obtained hash result into binary form, and intercept the first n bits as encoding features.

For the features service, protocol version and snapshots online rate, which have fewer feature values, they are encoded as [0, 1, 1] three-dimensional vectors using Binary Encoding. It follows that $B=\sum_{i=0}^{n-1}b_{i}2^{i}$, where *B* represents the value of the binary code, *b*_*i*_ represents the binary digit (either 0 or 1) at the *i*-th position, and *n* represents the number of bits in the binary code.

The original dataset has been transformed into a feature dataset suitable for machine learning.

**The fourth step**: cluster analysis.

We list 8 common unsupervised clustering algorithms and their computational complexity (see S2 Appendix). In this paper, the dataset has more features and large amount of data, so we choose three algorithms that have low computational complexity and are suitable for this dataset: MiniBatchKMeans clustering, Hierarchical clustering, and DBSCAN clustering. We experiment to select unsupervised clustering algorithms that can obtain better clustering results.

(1) MiniBatchKMeans: The MiniBatchKMeans algorithm is an optimized version of the KMeans algorithm for clustering on large data sets. Compared with the traditional KMeans algorithm, the MiniBatchKMeans algorithm is more suitable for this data set. Using this algorithm, a large amount of data can be processed faster and a similar clustering effect can be obtained. It follows that

$$\begin{array}{c} \underset{S_{1},\ldots ,S_{n}}{arg\;min}\;\sum_{i=1}^{n}\sum_{x\in S_{i}}\|x-\mu_{i}{\|}^{2}, \end{array}$$ (2)

where *n* represents the number of clusters, *S*_*i*_ represents the set of samples of the *i*-th cluster, *μ*_*i*_ represents the centroid of the *i*-th cluster, and ‖⋅‖ represents the Euclidean distance. The formula means that when clustering, all samples need to be divided into *n* clusters to minimize the distance between each sample and the centroid of its corresponding cluster.

The algorithm requires the number of clusters n to be clustered as input. But we don’t know how many clusters the data set should be divided into. We hope to get the answer through the effect of clustering. The Silhouette Coefficient is a measure of the similarity and dissimilarity of each data point to the cluster it belongs to. The value range of this indicator is between [-1, 1]. The closer the value is to 1, the better the clustering result is, and the closer the value is to -1, the worse the clustering result is. We cluster by increasing the value of n from 500 to 4500 and calculate the Silhouette Coefficient one by one. Finally, select the maximum value to determine the optimal number of clusters n. The Silhouette Coefficient follows $s=\frac{b-a}{max(a,b)}$, where *a* denotes the average distance between a sample and the other samples in its cluster.

To speed up the process, we increased the number of clusters classified in increments of 20 to 4500 to estimate the overall clustering effect. According to the local optimal result, the cluster number n of the global optimum is accurately calculated in increments of 1.

**Fig 11:**
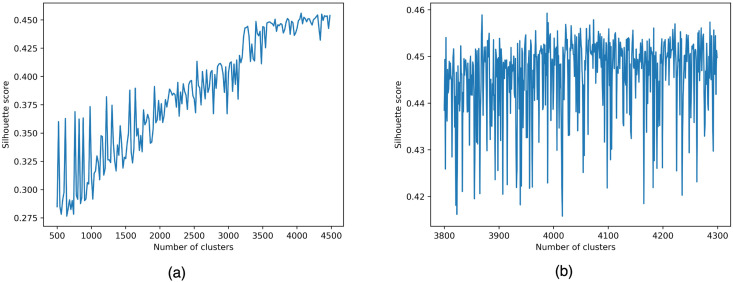
Silhouette coefficient variation curve with number of clusters (MiniBatchKMeans). In Fig 11, the horizontal axis represents the number of clusters of clusters and the vertical axis represents the silhouette coefficients. Fig 11(a) shows the overall change curve of the silhouette coefficients, and Fig 11(b) shows the exact change of the silhouette coefficients locally.

Through multiple rounds of experimental analysis, it is obtained that when the cluster value is 3988, random_state = 42(controls the random number seed of random initialization of each centroid, the random number is artificially specified, and 42 is considered a lucky number) The contour coefficient of each sample Highest. Finally, the optimal clustering result of the MiniBatchKMeans clustering algorithm is obtained (Fig 11).

(2) Hierarchical clustering. It is a clustering method based on the similarity or distance measurement between samples. Its main idea is to gradually merge similar or close samples into larger and larger clusters, until finally all samples are merged into one cluster or the preset stopping condition is reached.

The hierarchical clustering follows that

$$\begin{array}{c} D_{i,j}=\sqrt{\sum_{k=1}^{n}{\left(x_{i,k}-x_{j,k}\right)}^{2}}, \end{array}$$ (3)

where *D*_*i*,*j*_ represents the Euclidean distance between the *i*-th sample and the *j*-th sample. We also have

$$\begin{array}{c} d_{\text{new},n}=\frac{1}{k_{j}+k_{n}}(k_{j}d_{j,\text{new}}+k_{n}d_{n,\text{new}}+{\left(x_{j}-x_{n}\right)}^{T}\left(x_{j}-x_{n}\right)), \end{array}$$ (4)

where *d*_new,*n*_ represents the distance between the newly formed cluster and the *n*-th cluster; *k*_*j*_ and *k*_*n*_ represent the number of samples in the *j*-th cluster and *n*-th cluster, respectively; *d*_*j*,new_ and *d*_*n*,new_ represent the average distance among samples within the *j*-th and *n*-th clusters, respectively; ***x***_*j*_ and ***x***_*n*_ represent the mean vector of samples within the *j*-th and *n*-th clusters, respectively.

**Fig 12:**
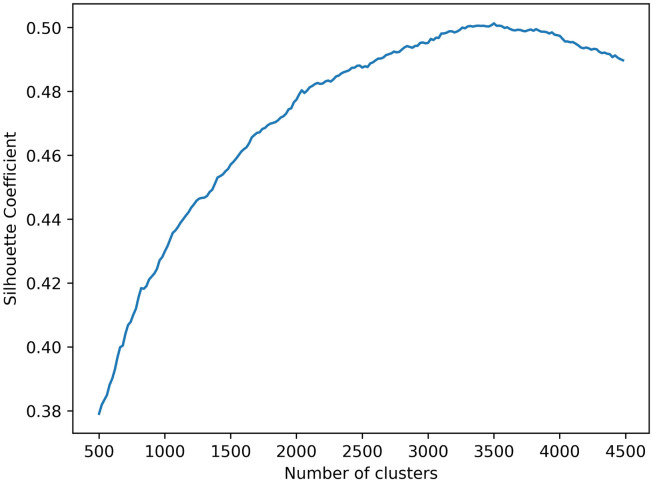
Silhouette coefficient curve with the number of clusters (Hierarchical clustering).

Hierarchical clustering also requires the number n of clusters to be entered. To unify the evaluation standard. We use the Silhouette Coefficient to evaluate the clustering results. We used the Hierarchical clustering algorithm many times, increasing the number of clusters n from 500 to 4500, and calculating the Silhouette Coefficient of each clustering result. Fig 12 shows the curve of the Silhouette Coefficient with the number of clusters. The clustering is best when the number of clusters n takes the value of 3500. This can be concluded that Hierarchical clustering is slightly better than MiniBatchKMeans.

(3) Density-Based Spatial Clustering of Applications with Noise (DBSCAN). It is a very popular clustering algorithm that automatically identifies areas with high density and divides them into a cluster. The DBSCAN does not need to specify the number of clusters in advance but determines the number and shape of clusters according to the density of the data. Therefore, the DBSCAN is more suitable for the scenarios used in this paper.

For any points *p* and *q* in a dataset, the *ϵ*-neighborhood of a point *p* is:

$$\begin{array}{c} N_{\epsilon }\left(p\right)=\left\{q|\text{dist}\left(p,q\right)\leq \epsilon \right\}, \end{array}$$ (5)

where dist(*p*, *q*) is the distance between points *p* and *q*, *p* and *q* are points in the dataset. *ϵ* is used to define the radius of the neighborhood. *N*_*ϵ*_(*p*) is the set of points in the *ϵ*-neighborhood of *p*. A point *p* is a core point if: |*N*_*ϵ*_(*p*)| ≥ MinPts, where MinPts represents the minimum number of objects in the neighborhood.

**Fig 13:**
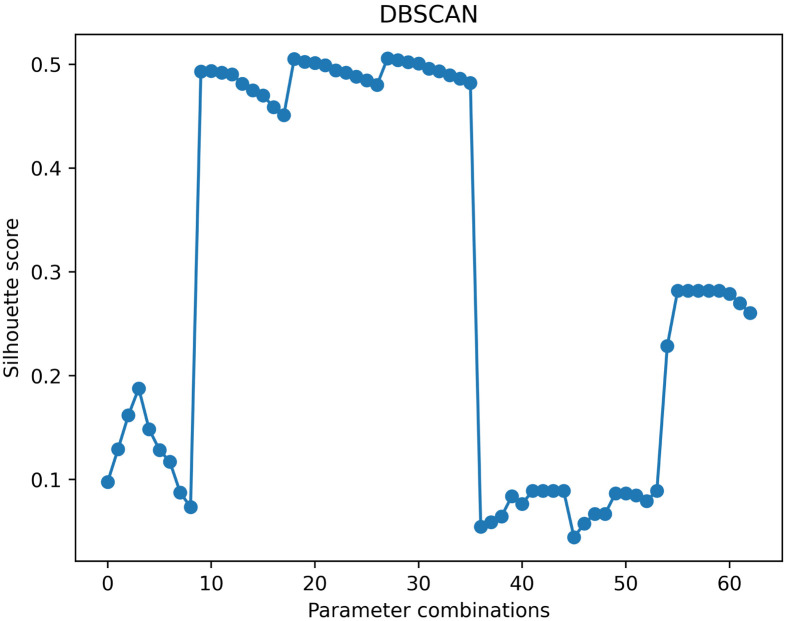
Silhouette coefficient curve with the number of clusters. Parameters combinations represent the Cartesian product of eps and min_samples.

We perform a clustering analysis of the above algorithms for Bitcoin nodes and compare them to arrive at the algorithm with the best clustering results. We use the Silhouette Coefficient to evaluate the clustering effect. Different from the previous two algorithms, the DBSCAN does not need to input the cluster number n of clustering but chooses eps and min_samples as variable parameters. The eps is the specified neighborhood radius used to determine the neighborhood of a point. The neighborhood of a point consists of all points contained in a circle with the point as the center and radius eps. min_samples refers to the minimum number of samples in the neighborhood. If the number of samples is less than min_samples, it is regarded as a noise point. Therefore, the DBSCAN can better reduce the impact of noise (single node) on the whole. We use a manual input parameter set: eps = [0.2, 0.5, 0.7, 0.9, 1.1, 1.3, 1.5], min_samples = [2, 3, 4, 5, 7, 9, 11, 13, 15]. And calculate its Cartesian product eps X min_samples for the input of DBSCAN. Parameter combinations refer to {(x,y) ∣ x ∈ *eps*, y ∈ *min*_*samples*}. The calculated Silhouette Coefficient varies with parameters as shown in Fig 13.

**Fig 14:**
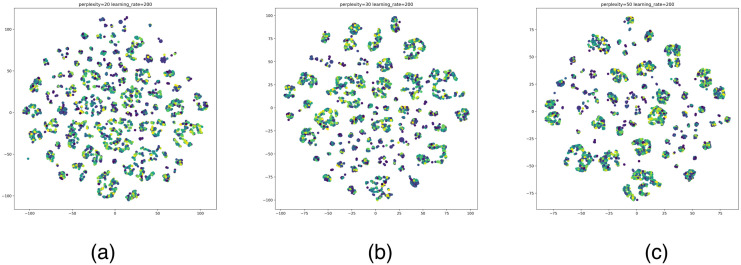
t-SNE visualization results of MiniBatchKMeans. (a)–(c) is the classification effects in the case of the optimal number of clusters. perplexity = 20, 30, 50, learning_rate = 200.

**Fig 15:**
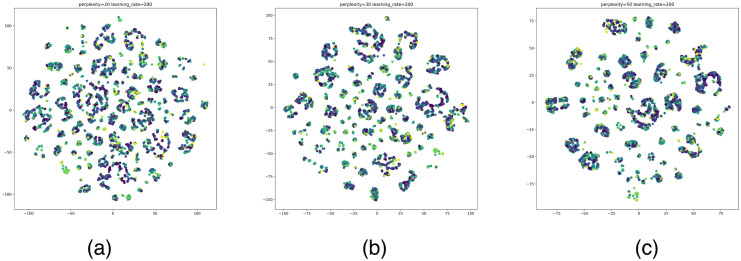
t-SNE visualization results of Hierarchical clustering. (a)–(c) is the classification effects in the case of the optimal number of clusters. perplexity = 20, 30, 50, learning_rate = 200.

**Fig 16:**
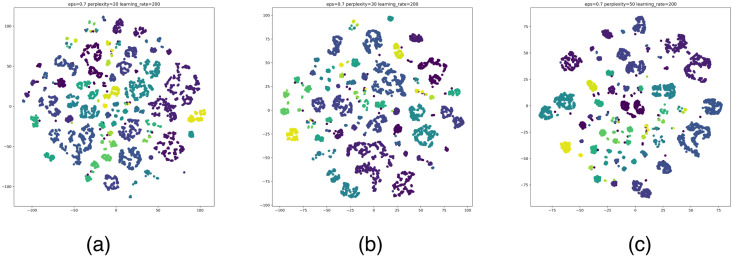
t-SNE visualization results of DBSCAN. (a)–(c) is the classification effects in the case of the optimal number of clusters. perplexity = 20, 30, 50, learning_rate = 200.

According to the clustering results of the above algorithms, the optimal number of clusters for MiniBatchKMeans is 3988, the optimal number of clusters for Hierarchical clustering is 3500, and the optimal number of clusters for DBSCAN is 115. The number of clusters of DBSCAN is significantly smaller than the former two. The DBSCAN removes multiple noisy data. To show the clustering effect of these three algorithms more intuitively, we visualize the optimal clustering results of MiniBatchKMeans, Hierarchical clustering, and DBSCAN with t-SNE. We use t-SNE to reduce the multidimensional features of the data, using a unified dimensionality reduction parameter. Draw category labels in different colors. To note: Perplexity is an important parameter in t-SNE to control the size of the neighborhood around each data point. It represents the number of nearest neighbor points considered in the dimensionality reduction process. Learning rate is another key parameter in t-SNE, which is used to control the step size or learning rate in the dimensionality reduction process. It determines the distance that the data points move in each iteration. The result is as shown in Figs 14–16.

According to the visual display of the best clustering results, in the DBSCAN clustering results, the nodes in the cluster (same color) are better gathered together. The clustering effect of MiniBatchKMeans and Hierarchical clustering is obviously overfitting, which causes the nodes (colored nodes) in the same cluster to be scattered. In summary, DBSCAN has a better effect on the clustering of Bitcoin node attributes.

## De-anonymization

For the clustering results presented in the previous subsection, this section focuses on de-anonymizing the nodes within the clusters. To achieve this, the approach followed involves accessing the web pages of the nodes within the clusters through their open HTTP/HTTPS services. Subsequently, based on the web page similarity and website functionality, we identify the deployers of the nodes. However, a few nodes were inaccessible due to network restrictions and firewalls. For the pages that were accessible and returned information (with a status code of 200), we obtained their titles for result determination. Please see Algorithm 3 for an overview of the process.

**Algorithm 3** Result Verification

**Input**: Cluster_IPs

**Output**: Status_Code,Site_Title

 **for** *IP* ∈ Cluster_IPs **do**

   *Protocol*, *Port* ⇐ Cyberspace_Search_Engine(*IP*)

3:   *Status*_*Code* ⇐ Get_Requests(*Protocol*://*IP*: *Port*)

   **if** *Status*_*Code* == 200 **then**

    *Site*_*Title* ⇐ Get_Title(*Protocol*://*IP*: *Port*)

6:   **end if**

 **end for**

**Fig 17:**
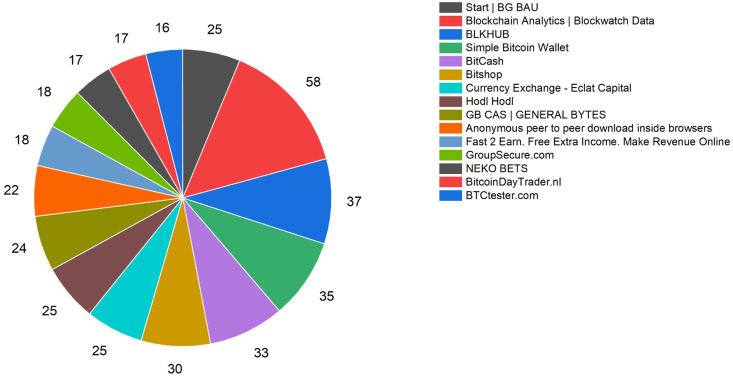
Teams corresponding to backbone nodes (partially).

Based on the clustering results presented in section Clustering Analysis of Bitcoin Addresses Based on Machine Learning, the dataset was divided into 115 clusters using DBSCAN. In order to validate the clustering effectiveness, the well-performing clusters were selected. Specifically, clusters with 16 or more nodes were chosen, and the top 15 clusters with a total of 400 nodes were identified. We manually reviewed the web pages of these nodes to determine their team affiliation. The results are shown in Fig 17.

The graph indicates that a significant number of nodes in the Bitcoin network are deployed by teams such as BlockChain Analytics, BLKHUB, and Simple Bitcoin Wallet. We manually queried the clustering results of each cluster to determine the teams associated with each one. We were able to identify 103 nodes with corresponding teams. This allows us to establish a link between the backbone nodes of the Bitcoin network and real-world organizations, achieving the objective of de-anonymizing Bitcoin nodes.

## Conclusion

The primary focus of this paper is to collect and analyze information related to Bitcoin nodes in order to explore the correlations between the backbone nodes of the Bitcoin network. More specifically, we identified and clustered the backbone nodes using both manual and machine learning algorithms, and then conducted a de-anonymization analysis on these nodes.

We continuously gathered Bitcoin node information between July 2021 and June 2022, during which period 127,613 unique IP addresses were detected on the Bitcoin network. We selected the stable, consistently online nodes to represent the backbone nodes of the Bitcoin network for analysis. By conducting statistical analysis and visualization of the backbone nodes’ geographic distribution, client versions, carriers, and service types, we gained a better understanding of the Bitcoin network. Our findings indicate a partial centralization of the Bitcoin network in several areas, such as geographical and client version centralization. We also observed the centralization of Bitcoin’s core functions, which provide a greater chance for the backbone nodes to earn rewards.

In this paper, we conducted clustering and de-anonymization analysis on the asset attributes of the backbone nodes and discovered their correlations. Our findings indicate that the backbone nodes can be divided into 115 clusters, and the IPs within each larger cluster exhibit significant clustering and similarity characteristics. In total, we identified 103 team affiliations among the backbone nodes.

By analyzing and de-anonymizing Bitcoin nodes, the public can gain a deeper understanding of Bitcoin. In future work, we aim to make the following improvements: 1. Create a better dataset and features. 2. Utilize more effective clustering algorithms. 3. Conduct analysis on the unreachable Bitcoin nodes.

## Supporting information

S1 Appendix Explanation of proper nouns. (DOCX)

S2 Appendix Comparison table of unsupervised clustering algorithms. (DOCX)

S1 Fig Bitcoin backbone node open web services. (DOCX)

S1 Table Complete table of bitcoin backbone node organization information. (DOCX)
