# Supplementary material for: Statistical and clustering analysis of attributes of Bitcoin backbone nodes
Source: PLoS One. 2023 Nov 8;18(11):e0292841. doi: 10.1371/journal.pone.0292841 (PMC10631630; doi:10.1371/journal.pone.0292841)
Supplement: S1 Appendix — (DOCX) [file pone.0292841.s001.docx]

**Supporting information**

**Is the Bitcoin network completely decentralized?**

Dawei Xu^1,2*^, Jiaqi Gao^1^, Liehuang Zhu^1^, Feng Gao^1^, Jian Zhao^2^

1 School of Cyberspace Security, Beijing Institute of Technology, Beijing, China

2 College of Cyber Security, Changchun University, Jilin, Changchun, China

S1 Appendix. Explanation of proper nouns

| **Bitcoin Network Protocol** | **Detailed description** |
| --- | --- |
| addr | broadcast its network address to the peer node. |
| getaddr | request other Bitcoin nodes to send a list of network addresses that they know about. |
| version | establish a connection between two bitcoin nodes and exchange information about each other. |
| versionack | Confirm that you have successfully received the"Version"message. |
| getblock | request data from other Bitcoin nodes for a specific block. |
| inv | broadcast and notify other nodes in the Bitcoin network about the existence of specific data objects. |
| getdata | request details about a specific data object from other Bitcoin nodes. |
| getheaders | request other Bitcoin nodes to send block headers. |
| headers | transfer information about multiple block heads across the Bitcoin network. |
