# Supplementary material for: Statistical and clustering analysis of attributes of Bitcoin backbone nodes
Source: PLoS One. 2023 Nov 8;18(11):e0292841. doi: 10.1371/journal.pone.0292841 (PMC10631630; doi:10.1371/journal.pone.0292841)
Supplement: S2 Appendix — (DOCX) [file pone.0292841.s002.docx]

**Supporting information**

**Is the Bitcoin network completely decentralized?**

Dawei Xu^1,2*^, Jiaqi Gao^1^, Liehuang Zhu^1^, Feng Gao^1^, Jian Zhao^2^

1 School of Cyberspace Security, Beijing Institute of Technology, Beijing, China

2 College of Cyber Security, Changchun University, Jilin, Changchun, China

**S2 Appendix. Comparison table of unsupervised clustering algorithms**

| **Algorithm** | **Time complexity** | **Preset number of clusters** | **Handle irregularly shaped clusters** |
| --- | --- | --- | --- |
| K-means | O(I · K · N · d) | ✓ | ✕ |
| Hierarchy clustering | O(n^3) – O(n^2 log n) | ✕ | ✓ |
| DBSCAN | O(N · log(N)) | ✕ | ✓ |
| Gaussian Mixture Model | O(I · N · K · d^2) | ✓ | ✕ |
| Spectral clustering | O(N^2 · d) | ✓ | ✓ |
| Agglomerative clustering | O(N^3) | X | ✓ |
| OPTICS | O(N^2 · log(N)) | X | ✓ |
| MiniBatchKMeans | O(I · K · batch_size · d) | ✓ | ✕ |

K: number of clusters. N: number of data points. d: dimension of the data points. batch_size: the size of a small batch. I: Number of iterations.
