# Supplementary material for: Statistical and clustering analysis of attributes of Bitcoin backbone nodes
Source: PLoS One. 2023 Nov 8;18(11):e0292841. doi: 10.1371/journal.pone.0292841 (PMC10631630; doi:10.1371/journal.pone.0292841)

**Supporting information**

**Is the Bitcoin network completely decentralized?**

Dawei Xu^1,2*^, Jiaqi Gao^1^, Liehuang Zhu^1^, Feng Gao^1^, Jian Zhao^2^

1 School of Cyberspace Security, Beijing Institute of Technology, Beijing, China

2 College of Cyber Security, Changchun University, Jilin, Changchun, China

**S1 Fig. Wallet website.** Access the backbone node's IP address + port through your browser to get the node's functionality open in the real world. As you can tell from the content of the screenshot, this bitcoin backbone node is open for bitcoin wallet services.


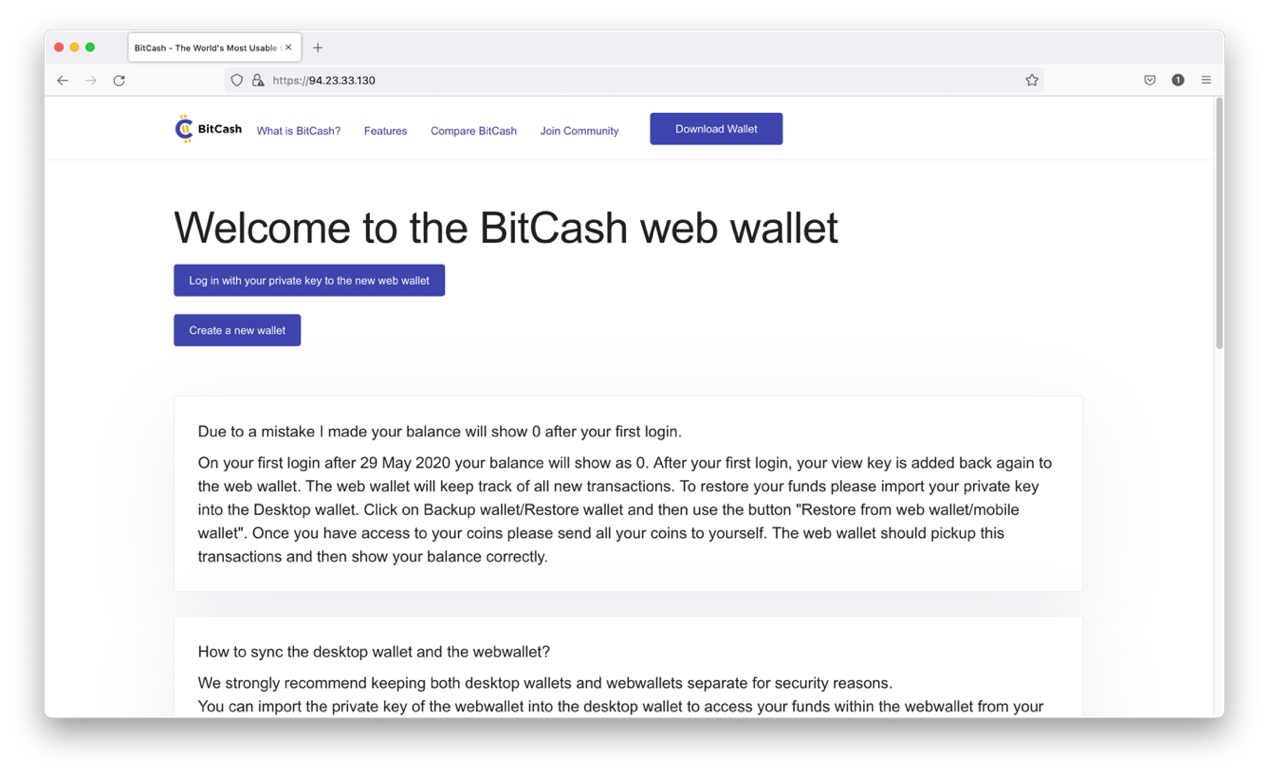


**S2 Fig.** **Bitcoin monitoring site.** Based on the content of this website, it is clear that this node runs the bitcoin monitoring website. It includes the number of bitcoin nodes, mining difficulties, etc.


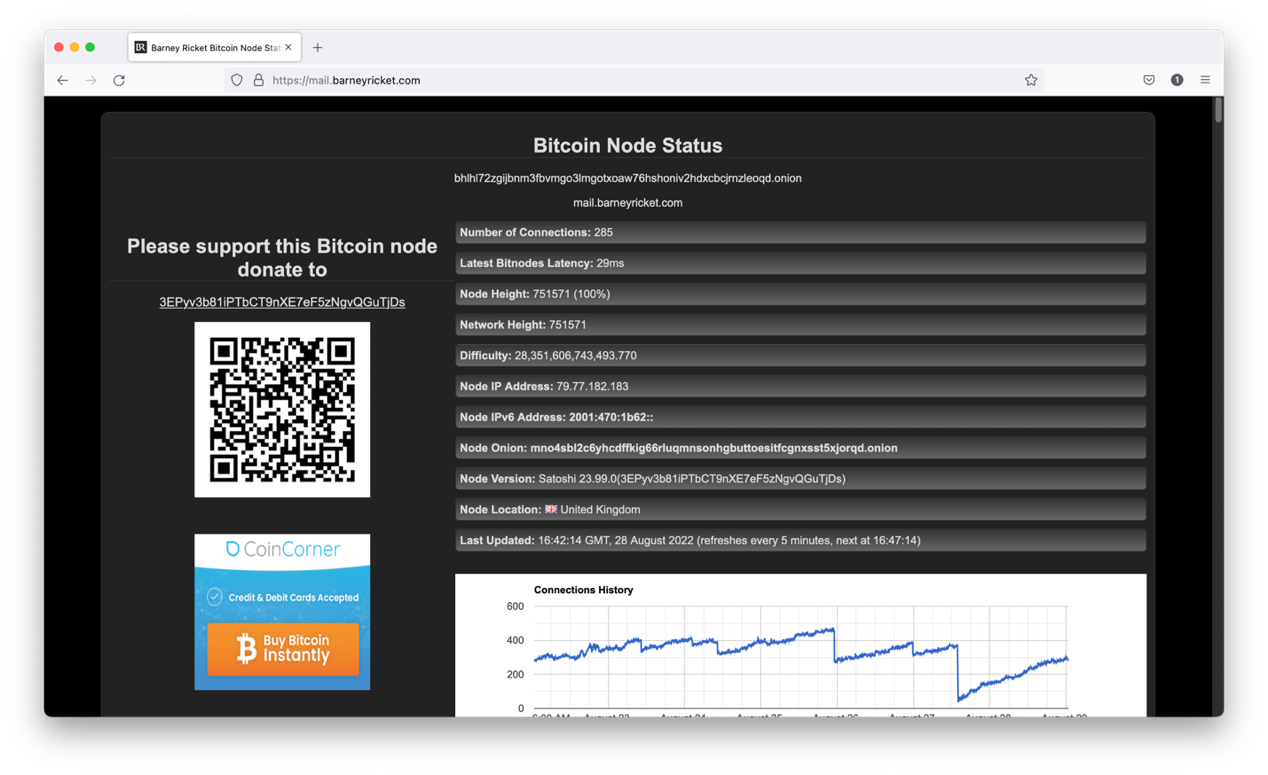


**S3 Fig. Bitcoin transaction monitoring issues.** Based on the content of this website, it is known that this node runs a website that monitors bitcoin transactions. This includes the time of each transaction as well as the hash value, block header number, etc.


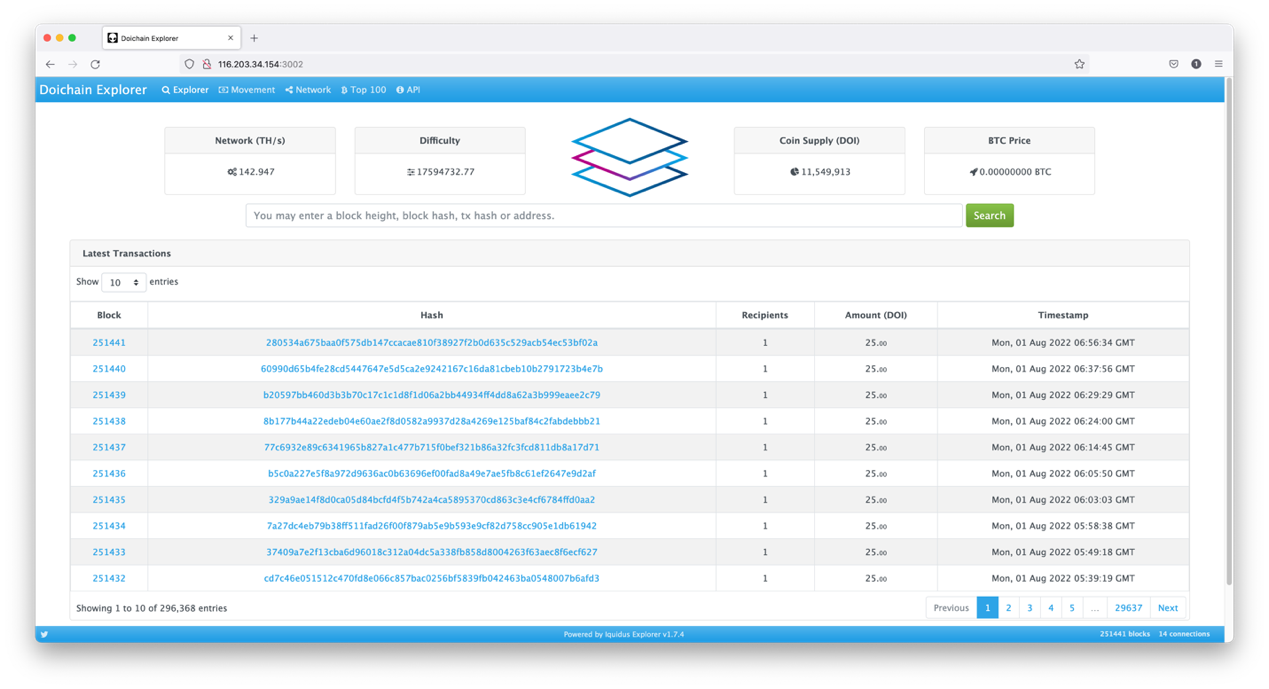


**S4 Fig.** **Bitcoin file download service.** According to the content of this website, it is available to download bitcoin-related file data from this node.


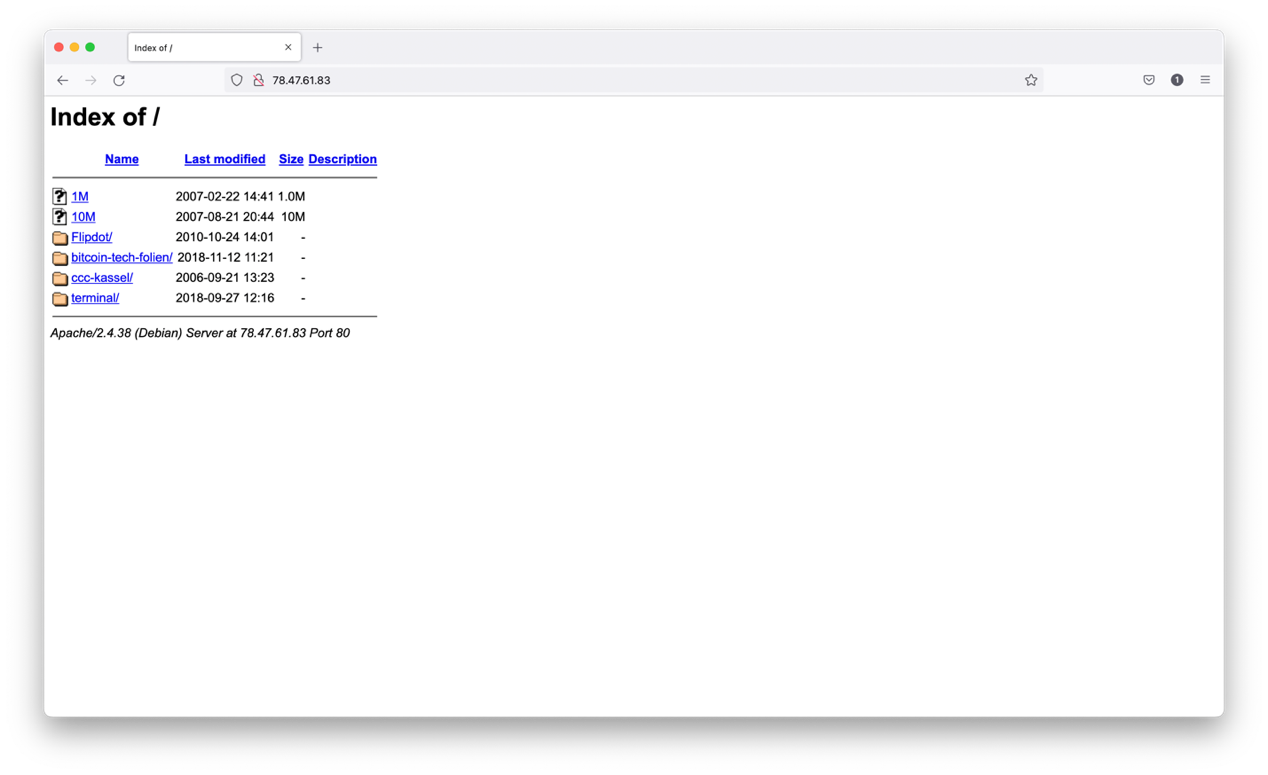


**S5 Fig. Mining pool performance testing and mining pool information.** According to the content of this website, this node collects information about the currently available mining pools, as well as their arithmetic power and other attributes.


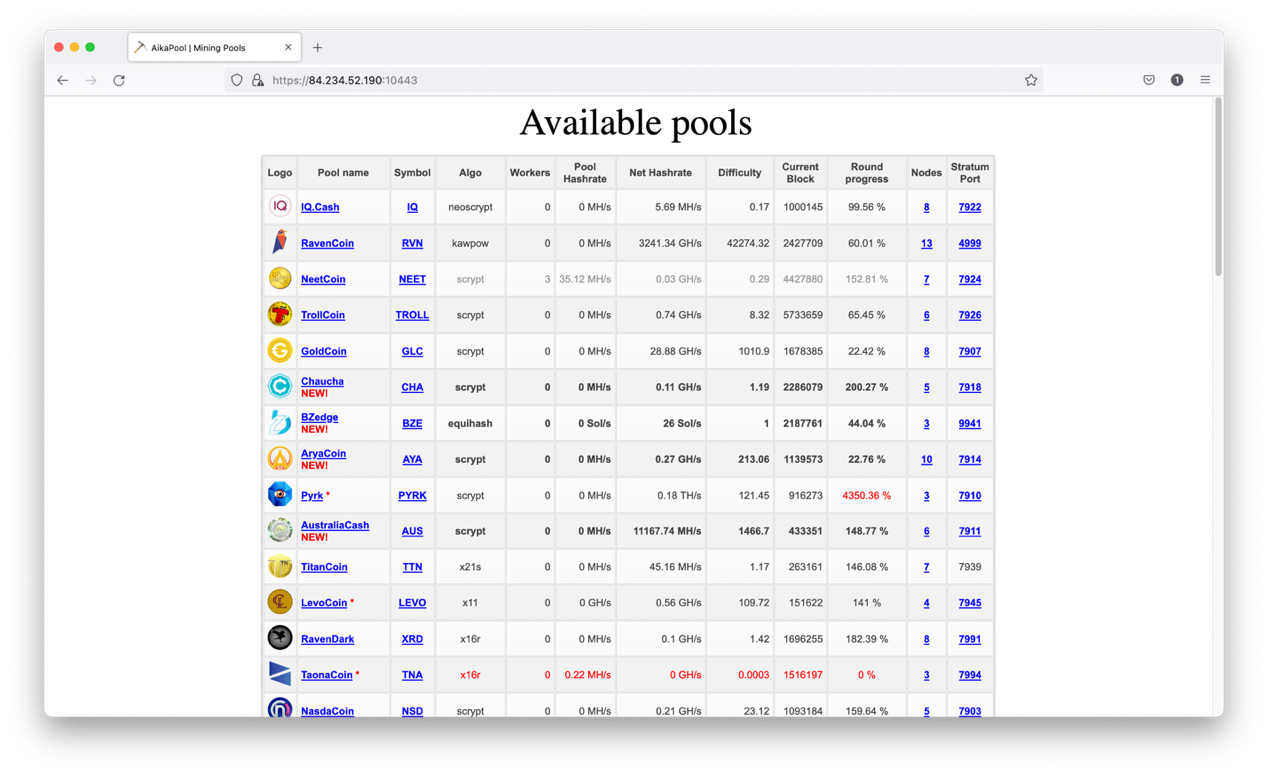


**S6 Fig. User backend login interfaces.** This site is the backend login screen for doichain.


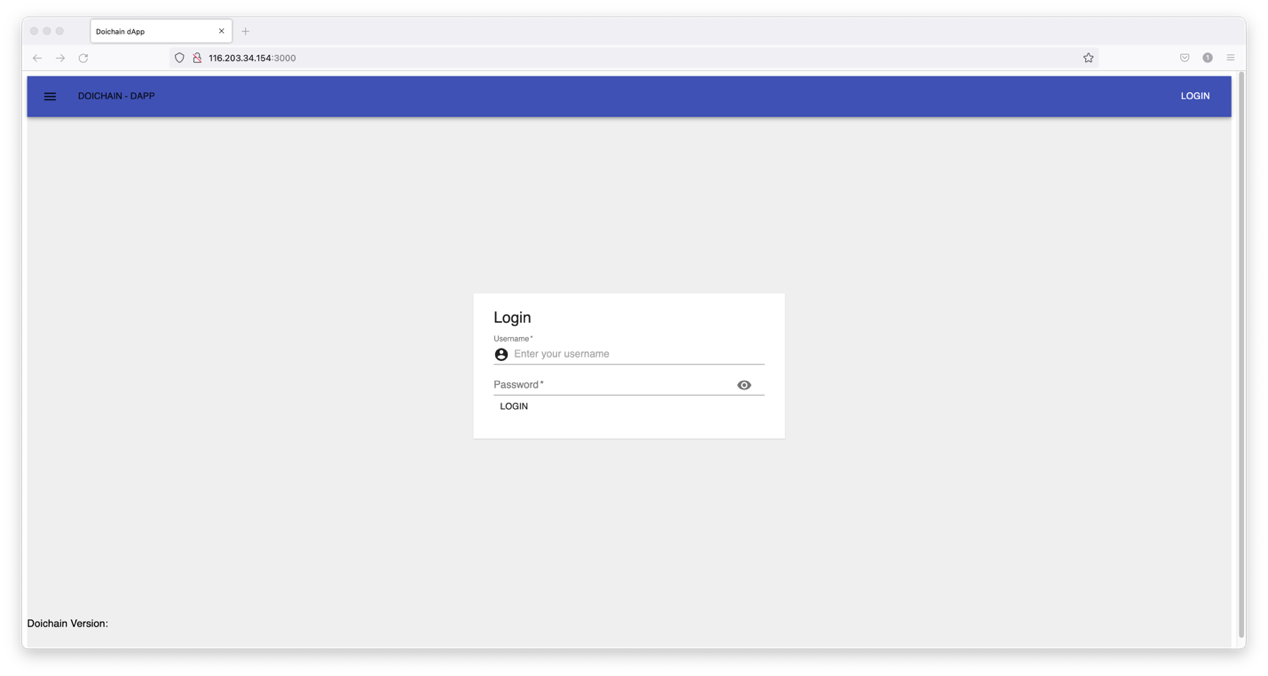


**S7 Fig. Mining information interface.** This node is running a bitcoin miner. The screenshot interface shows the relevant performance parameters of the miner.


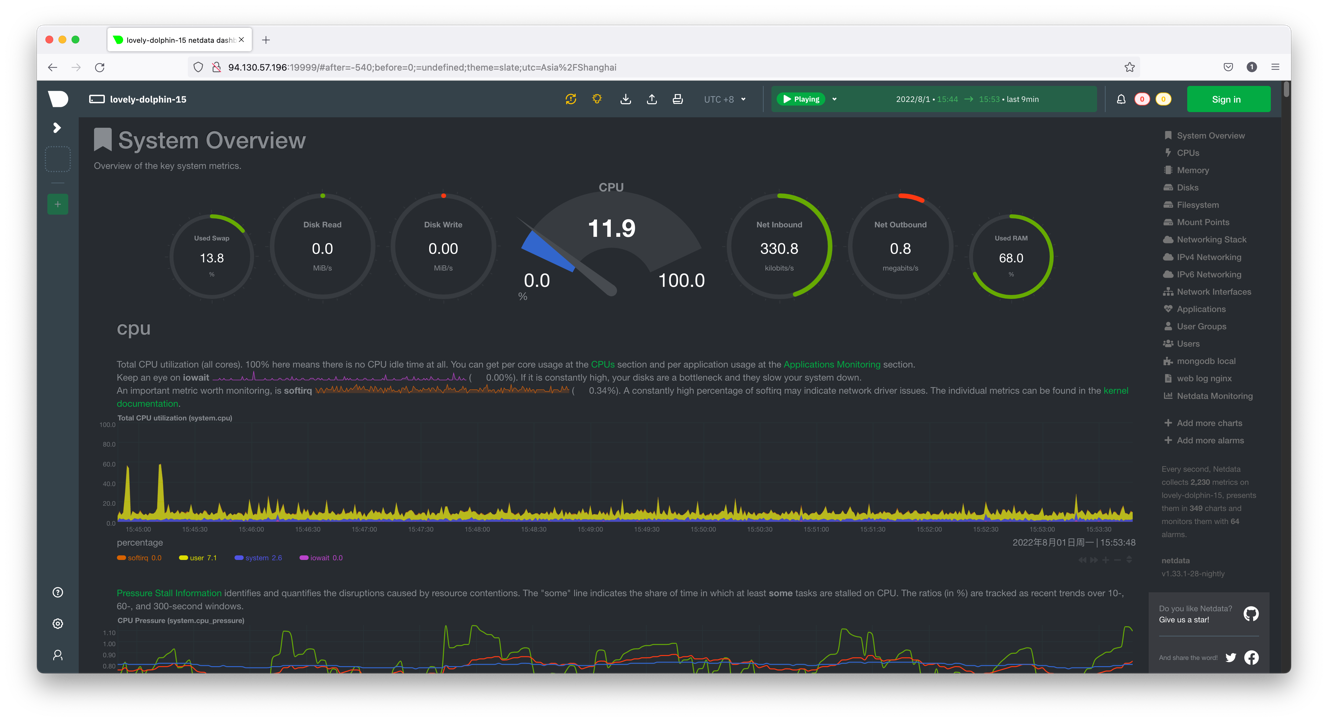

Supplement: S1 Fig — (DOCX) [file pone.0292841.s003.docx]
