# Supplementary material for: Statistical and clustering analysis of attributes of Bitcoin backbone nodes
Source: PLoS One. 2023 Nov 8;18(11):e0292841. doi: 10.1371/journal.pone.0292841 (PMC10631630; doi:10.1371/journal.pone.0292841)
Supplement: S1 Table — (DOCX) [file pone.0292841.s004.docx]

**Supporting information**

**Is the Bitcoin network completely decentralized?**

Dawei Xu^1,2*^, Jiaqi Gao^1^, Liehuang Zhu^1^, Feng Gao^1^, Jian Zhao^2^

1 School of Cyberspace Security, Beijing Institute of Technology, Beijing, China

2 College of Cyber Security, Changchun University, Jilin, Changchun, China

**S1 Table. Results of clustering analysis of abnormal port numbers**

| ip | tag | port | lon | lat | organization |
| --- | --- | --- | --- | --- | --- |
| 221.219.97.105 | 1 | 2001 | 116.397459 | 39.938884 | China Unicom Beijing Province Network |
| 221.219.102.228 | 1 | 2001 | 116.397459 | 39.938884 | China Unicom Beijing Province Network |
| 63.32.65.205 | 2 | 5001 | -6.26031 | 53.349805 | Amazon.com, Inc. |
| 54.76.224.236 | 2 | 5001 | -6.26031 | 53.349805 | Amazon.com, Inc. |
| 34.248.223.238 | 2 | 5001 | -6.26031 | 53.349805 | Amazon.com, Inc. |
| 34.252.91.3 | 2 | 5001 | -6.26031 | 53.349805 | Amazon.com, Inc. |
| 54.195.171.69 | 2 | 5001 | -6.26031 | 53.349805 | Amazon.com, Inc. |
| 82.193.103.110 | 3 | 6333 | 30.5234 | 50.4501 | PJSC "Industrial Media Network" |
| 144.91.66.137 | 4 | 6455 | 11.07349 | 49.454342 | Contabo GmbH |
| 173.212.243.27 | 5 | 7662 | 11.07349 | 49.454342 | Contabo GmbH |
| 54.36.62.114 | 6 | 8253 | 2.142824 | 51.002837 | OVH SAS |
| 213.239.232.113 | 7 | 8332 | 11.07349 | 49.454342 | Hetzner Online GmbH |
| 163.172.142.149 | 7 | 8332 | 2.352222 | 48.856614 | ONLINE S.A.S. |
| 162.55.101.8 | 7 | 8332 | 11.07349 | 49.454342 | Hetzner Online GmbH |
| 162.55.7.43 | 7 | 8332 | 12.364975 | 50.475005 | Hetzner Online GmbH |
| 188.32.14.31 | 8 | 8334 | 37.605061 | 55.741638 | PJSC Rostelecom |
| 88.99.149.100 | 9 | 8335 | 12.364975 | 50.475005 | Hetzner Online GmbH |
| 94.130.35.227 | 9 | 8335 | 12.364975 | 50.475005 | Hetzner Online GmbH |
| 13.82.198.161 | 9 | 8335 | -77.429129 | 39.006699 | Microsoft Corporation |
| 73.200.139.162 | 9 | 8335 | -77.61388 | 38.795671 | Comcast Cable Communications, LLC |
| 66.151.242.154 | 9 | 8335 | -87.629798 | 41.878114 | Dedicated.com |
| 31.25.241.224 | 9 | 8335 | 60.605703 | 56.838926 | data-centr ekaterinburg OOO |
| 94.19.132.255 | 9 | 8335 | 30.335099 | 59.93428 | SkyNet Ltd. |
| 60.251.129.61 | 10 | 8336 | 121.565427 | 25.032964 | Chunghwa Telecom Co., Ltd. |
| 5.186.60.13 | 10 | 8336 | 9.51695 | 56.276089 | FIBIA P/S |
| 51.255.87.121 | 11 | 8339 | 2.142824 | 51.002837 | OVH SAS |
| 148.251.1.20 | 12 | 8343 | 12.364975 | 50.475005 | Hetzner Online GmbH |
| 193.42.110.30 | 12 | 8343 | 4.89319 | 52.373119 | 3W Infra B.V. |
| 20.184.15.116 | 12 | 8433 | 103.819836 | 1.352083 | Microsoft Corporation |
| 46.166.128.226 | 13 | 8444 | 4.89319 | 52.373119 | NForce Entertainment B.V. |
| 142.44.142.126 | 13 | 8444 | -73.877903 | 45.315081 | OVH SAS |
| 159.250.153.179 | 13 | 8444 | -81.515754 | 27.664827 | Atlantic Broadband Finance, LLC |
| 85.10.201.29 | 14 | 8446 | 11.07349 | 49.454342 | Hetzner Online GmbH |
| 116.203.200.229 | 14 | 8446 | 11.07349 | 49.454342 | Hetzner Online GmbH |
| 212.90.58.56 | 15 | 8512 | 30.5234 | 50.4501 | Scientific-Production Enterprise Information Technologies Ltd |
| 172.105.179.145 | 16 | 8555 | 151.209295 | -33.868819 | Linode, LLC |
| 172.105.180.126 | 16 | 8555 | 151.209295 | -33.868819 | Linode, LLC |
| 65.21.142.118 | 16 | 8555 | 24.932581 | 60.171162 | Hetzner Online GmbH |
| 24.55.154.69 | 16 | 8555 | -75.817976 | 40.453983 | PenTeleData Inc. |
| 51.255.64.196 | 17 | 8800 | 3.18125 | 50.688438 | OVH SAS |
| 107.190.47.97 | 18 | 8833 | -73.625122 | 45.536491 | TekSavvy Solutions, Inc. |
| 190.2.138.4 | 19 | 9000 | 4.199261 | 51.995911 | WorldStream B.V. |
| 49.12.126.6 | 20 | 9001 | 12.364975 | 50.475005 | Hetzner Online GmbH |
| 49.12.163.214 | 20 | 9001 | 11.07349 | 49.454342 | Hetzner Online GmbH |
| 94.130.64.182 | 20 | 9001 | 12.364975 | 50.475005 | Hetzner Online GmbH |
| 78.46.196.248 | 20 | 9001 | 12.364975 | 50.475005 | Hetzner Online GmbH |
| 212.99.226.35 | 21 | 9020 | 12.549326 | 55.675181 | Sagitta ApS |
| 35.163.48.30 | 22 | 9091 | -122.65872 | 45.512231 | Amazon.com, Inc. |
| 49.12.163.214 | 23 | 9199 | 11.07349 | 49.454342 | Hetzner Online GmbH |
| 94.130.64.182 | 23 | 9199 | 12.364975 | 50.475005 | Hetzner Online GmbH |
| 75.119.132.199 | 24 | 9333 | 6.77604 | 51.21563 | Contabo GmbH |
| 37.59.53.126 | 24 | 9333 | 3.18125 | 50.688438 | OVH SAS |
| 46.165.221.209 | 24 | 9333 | 8.68341 | 50.11208 | Leaseweb Deutschland GmbH |
| 81.21.86.157 | 24 | 9333 | 47.687069 | 40.15134 | Ultel LLC |
| 136.144.215.219 | 25 | 10100 | 4.477733 | 51.92442 | Transip B.V. |
| 75.119.132.199 | 26 | 10333 | 6.77604 | 51.21563 | Contabo GmbH |
| 92.255.170.159 | 27 | 11080 | 65.572 | 57.174086 | JSC "ER-Telecom Holding" |
| 85.15.179.171 | 27 | 11080 | 66.948278 | 56.963439 | PJSC Rostelecom |
| 91.121.221.92 | 27 | 11080 | 3.18125 | 50.688438 | OVH SAS |
| 91.206.16.214 | 27 | 11080 | 65.572 | 57.174086 | Delta Telesystems Ltd. |
| 79.98.159.7 | 28 | 11333 | 15.32814 | 50.605157 | NETAIR, s.r.o. |
| 95.216.242.53 | 29 | 12853 | 24.932581 | 60.171162 | Hetzner Online GmbH |
| 167.114.156.121 | 30 | 14333 | -73.877903 | 45.315081 | OVH SAS |
| 139.162.22.75 | 31 | 18222 | 103.819836 | 1.352083 | Linode, LLC |
| 47.100.162.210 | 31 | 18332 | 121.476753 | 31.224349 | Hangzhou Alibaba Advertising Co.,Ltd. |
| 207.154.230.25 | 32 | 18333 | 8.68341 | 50.11208 | DigitalOcean, LLC |
| 138.201.78.28 | 32 | 18333 | 12.364975 | 50.475005 | Hetzner Online GmbH |
| 135.181.137.135 | 32 | 18333 | 24.932581 | 60.171162 | Hetzner Online GmbH |
| 35.175.179.123 | 32 | 18333 | -77.487442 | 39.043757 | Amazon.com, Inc. |
| 95.216.15.164 | 32 | 18333 | 24.932581 | 60.171162 | Hetzner Online GmbH |
| 46.30.189.41 | 33 | 19911 | 8.68341 | 50.11208 | diva-e Datacenters GmbH |
| 3.0.108.49 | 34 | 20008 | 103.819836 | 1.352083 | Amazon.com, Inc. |
| 87.120.8.5 | 34 | 20008 | 23.321867 | 42.697708 | Neterra Ltd. |
| 174.138.58.171 | 34 | 20008 | -74.005941 | 40.712784 | DigitalOcean, LLC |
| 134.209.234.186 | 34 | 20008 | 8.68341 | 50.11208 | DigitalOcean, LLC |
| 45.63.10.52 | 34 | 20008 | -74.464286 | 40.554887 | The Constant Company, LLC |
| 107.23.66.252 | 34 | 20008 | -77.487442 | 39.043757 | Amazon.com, Inc. |
| 47.96.119.168 | 34 | 20018 | 120.165024 | 30.252501 | Hangzhou Alibaba Advertising Co.,Ltd. |
| 185.15.92.18 | 35 | 20993 | 8.68341 | 50.11208 | AirAccess GmbH trading as 'Lightup Network Solutions GmbH & Co. KG |
| 158.69.227.12 | 36 | 28633 | -73.877903 | 45.315081 | OVH SAS |
| 158.69.227.12 | 37 | 28643 | -73.877903 | 45.315081 | OVH SAS |
| 51.138.4.135 | 38 | 30001 | 4.89319 | 52.373119 | Microsoft Corporation |
| 51.136.85.64 | 38 | 30001 | 4.89319 | 52.373119 | Microsoft Corporation |
| 51.136.85.64 | 39 | 30002 | 4.89319 | 52.373119 | Microsoft Corporation |
| 51.138.4.135 | 39 | 30002 | 4.89319 | 52.373119 | Microsoft Corporation |
| 178.63.69.234 | 40 | 30200 | 12.364975 | 50.475005 | Hetzner Online GmbH |
| 138.68.8.225 | 40 | 30200 | -122.41942 | 37.77493 | DigitalOcean, LLC |
| 144.76.71.116 | 41 | 33354 | 12.364975 | 50.475005 | Hetzner Online GmbH |
| 80.61.232.204 | 42 | 39388 | 5.32986 | 52.1082 | KPN B.V. |
| 52.14.104.124 | 43 | 48333 | -83.114077 | 40.099229 | Amazon.com, Inc. |
| 185.254.75.50 | 44 | 54895 | 6.77604 | 51.21563 | xTom GmbH |
| 51.38.57.53 | 45 | 55859 | 2.142824 | 51.002837 | OVH SAS |
| 45.32.109.115 | 46 | 56792 | 103.819836 | 1.352083 | The Constant Company, LLC |
| 109.236.81.138 | 47 | 58333 | 4.199261 | 51.995911 | WorldStream B.V. |
| 80.88.172.227 | 48 | 64264 | 9.181873 | 45.458626 | MYNET S.R.L. |

**S2 Table. Bitcoin backbone node clustering and de-anonymization results**

| IP | URL | Count | Web site title |
| --- | --- | --- | --- |
| 144.76.40.171 | https://144.76.40.171:443 | 25 | <title>Start \| BG BAU - Berufsgenossenschaft der Bauwirtschaft</title> |
| 94.130.71.31 | https://94.130.71.31:443 | 58 | <title ng-bind="site.title()">Blockchain Analytics \| Blockwatch Data</title> |
| 51.254.199.134 | [https://51.254.199.134:443](https://51.254.199.134/) | 37 | <title>BLKHUB</title> |
| 5.9.83.143 | [https://5.9.83.143:443](https://5.9.83.143/) | 35 | <title>Simple Bitcoin Wallet</title> |
| 94.23.33.130 | https://94.23.33.130:443 | 33 | <title>BitCash - The World's Most Usable Cryptocurrency</title> |
| 161.97.131.168 | [http://161.97.131.168:80](http://161.97.131.168/) | 30 | <title>Contact - Bitshop</title> |
| 94.130.108.26 | [http://94.130.108.26:80](http://94.130.108.26/) | 25 | <title>Currency Exchange - Eclat Capital</title> |
| 178.63.16.7 | [http://178.63.16.7:8080](http://178.63.16.7:8080/) | 25 | <title>start [Hodl Hodl]</title> |
| 134.209.74.26 | [https://134.209.74.26:7777](https://134.209.74.26:7777/) | 24 | <title>GB CAS \| GENERAL BYTES - Crypto Application Server</title> |
| 37.59.47.27 | [https://37.59.47.27:443](https://37.59.47.27/) | 22 | <title>Anonymous peer to peer download inside browsers and distributed database inside browsers, compatible with torrents but encrypted and untrackable</title> |
| 5.135.178.216 | [https://5.135.178.216:443](https://5.135.178.216/) | 18 | <title>Fast 2 Earn. Free Extra Income. Make Revenue Online</title> |
| 95.216.226.218 | https://95.216.226.218:443 | 18 | <title>GroupSecure.com</title> |
| 5.9.144.250 | [https://5.9.144.250:443](https://5.9.144.250/) | 17 | <title>NEKO BETS \| Crypto's Fastest Growing Casino</title> |
| 217.104.75.11 | [https://217.104.75.11:443](https://217.104.75.11/) | 17 | <title>BitcoinDayTrader.nl</title> |
| 5.189.187.89 | [http://5.189.187.89:80](http://5.189.187.89/) | 16 | <title>BTCtester.com Bitcoin Forensics 鈥 Follow Bitcoins into the blockchain</title> |
| 91.121.116.107 | [http://91.121.116.107:80](http://91.121.116.107/) | 16 | <title>phpLDAPadmin (1.2.3) - </title> |
| 96.237.231.17 | https://96.237.231.17:443 | 16 | <title>Bloggy McBlogFace</title> |
| 5.9.5.135 | [https://5.9.5.135:443](https://5.9.5.135/) | 14 | <title>hito wallet</title> |
| 85.214.103.48 | [http://85.214.103.48:3001](http://85.214.103.48:3001/) | 14 | <title>A Light To Remember - Gallery</title> |
| 95.216.96.93 | [http://95.216.96.93:80](http://95.216.96.93/) | 13 | <title>CAD-KAS Software - PDF Editor - Shareware download page</title> |
| 54.159.193.149 | [https://54.159.193.149:8443](https://54.159.193.149:8443/) | 13 | <title>Lightning Terminal</title> |
| 82.71.47.216 | [http://82.71.47.216:80](http://82.71.47.216/) | 13 | <title>Chataigne!</title> |
| 45.15.61.233 | [http://45.15.61.233:80](http://45.15.61.233/) | 12 | <title>Nextcloud </title> |
| 167.114.34.134 | http://167.114.34.134:80 | 12 | <title>The Buckmaster Institute, Inc. - Productions</title> |
| 188.68.53.44 | http://188.68.53.44:80 | 12 | <title>188.68.53.44 - Shorena's full Bitcoin node v2</title> |
| 80.240.129.221 | https://80.240.129.221:443 | 11 | <title>Crypto.bg</title> |
| 64.4.160.13 | http://64.4.160.13:80 | 11 | <title>RhinoAnt.com \| Pool Dashboard</title> |
| 99.36.167.158 | http://99.36.167.158:8181 | 11 | <title>Tautulli - Login</title> |
| 159.65.57.197 | https://159.65.57.197:443 | 11 | <title>e-senseREHA</title> |
| 95.217.38.60 | [http://95.217.38.60:19999](http://95.217.38.60:19999/) | 10 | <title>netdata dashboard</title> |
| 78.102.79.63 | http://78.102.79.63:80 | 10 | <title>lucaash.com</title> |
| 195.154.187.6 | [http://195.154.187.6:80](http://195.154.187.6/) | 10 | <title>WINDICE</title> |
